# A novel methoxydotrophic metabolism discovered in the hyperthermophilic archaeon *Archaeoglobus fulgidus*

**DOI:** 10.1111/1462-2920.15546

**Published:** 2021-05-05

**Authors:** Cornelia U. Welte, Rob de Graaf, Paula Dalcin Martins, Robert S. Jansen, Mike S. M. Jetten, Julia M. Kurth

**Affiliations:** ^1^ Department of Microbiology, Institute for Water and Wetland Research Radboud University Heyendaalseweg 135 Nijmegen 6525 AJ The Netherlands; ^2^ Netherlands Earth System Science Center Utrecht University, Heidelberglaan 2 Utrecht 3584 CS The Netherlands; ^3^ Soehngen Institute of Anaerobic Microbiology Radboud University, Heyendaalseweg 135 Nijmegen 6525 AJ The Netherlands

## Abstract

Methoxylated aromatic compounds (MACs) are important components of lignin found in significant amounts in the subsurface. Recently, the methanogenic archaeon *Methermicoccus shengliensis* was shown to be able to use a variety of MACs during methoxydotrophic growth. After a molecular survey, we found that the hyperthermophilic non‐methanogenic archaeon *Archaeoglobus fulgidus* also encodes genes for a bacterial‐like demethoxylation system. In this study, we performed growth and metabolite analysis, and used transcriptomics to investigate the response of *A. fulgidus* during growth on MACs in comparison to growth on lactate. We observed that *A. fulgidus* converts MACs to their hydroxylated derivatives with CO_2_ as the main product and sulfate as electron acceptor. Furthermore, we could show that MACs improve the growth of *A. fulgidus* in the presence of organic substrates such as lactate. We also found evidence that other archaea such as Bathyarchaeota, Lokiarchaeota, Verstraetearchaeota, Korarchaeota, Helarchaeota and Nezhaarchaeota encode a demethoxylation system. In summary, we here describe the first non‐methanogenic archaeon with the ability to grow on MACs indicating that methoxydotrophic archaea might play a so far underestimated role in the global carbon cycle.

## Introduction

Aromatic compounds are produced by plants, animals and microorganisms and are therefore quite abundant on earth. The polyaromatic compound lignin can be found in significant amounts in the subsurface. Lignin is a major component of photosynthetic biomass and makes up approximately 25% of the dry weight of vascular plants (Zeikus, 1981). It is further estimated that approximately one third of the organic matter present in marine sediments is of terrestrial origin (Burdige, 2005). Overall, lignin analysis indicated that peat and coastal marine sediments contained about 20%–50% of recognizable vascular plant carbon and soils of offshore marine sediments about 0%–10% (Ertel and Hedges, 1984).

There are many possibilities for substitutions on the aromatic ring and one of those possible ring modifications is the addition of methoxy groups resulting in so‐called methoxylated aromatic compounds. Lignin contains about 3% methoxy groups respectively (Lee *et al*., 2019) and methoxylated aromatic compounds as major components of lignin are quite abundant in natural environments (Hedges *et al*., 1982; Colberg, 1988). Alkyl‐methoxyphenols have been found to be typical pyrolysis products of lignin‐derived materials (Salmon *et al*., 1997). It is well known that methoxylated aromatic compounds can be degraded by bacteria. Already in 1979, it was shown that methoxylated aromatic compounds can be converted to methane by the syntrophic association of several anaerobic microorganisms (Healy and Young, 1979). Acetogenic bacteria were the first anaerobes discovered to use methoxylated aromatic compounds for energy conservation (Bache and Pfennig, 1981) via conversion of the methyl group to acetate in the acetyl‐CoA (Wood‐Ljungdahl) pathway. Some bacteria such as *Sporobacter termitidis* (Grech‐Mora *et al*., 1996), *Sporobacter olearium* (Mechichi *et al*., 1999) and *Parasporobacterium paucivorans* (Lomans *et al*., 2001) are able to cleave the aromatic ring after *O*‐demethylation and convert methoxylated aromatic compounds to acetate, methanethiol or dimethylsulfide. *O*‐demethylation is an essential step preceding aromatic ring cleavage. Next to these ring‐cleaving methoxydotrophic acetogens, several acetogens have been described to *O*‐demethylate methoxylated aromatic compounds and to release the corresponding hydroxylated derivatives into the environment without cleaving the aromatic ring. The methoxy group is transferred into the acetyl‐CoA pathway resulting in formation of products such as acetate. Examples of this type of methoxydotrophic acetogens are *Sporomusa termitida* (Breznak *et al*., 1988), *Clostridium thermoaceticum* (Wu *et al*., 1988) and *Acetobacterium woodii* (Bache and Pfennig, 1981). Acetogens use two methyltransferases, one corrinoid protein and one activating enzyme that recycles the corrinoid protein for transfer of the methyl group from methoxylated compounds (Kaufmann *et al*., 1997). These enzymes catalyse the *O*‐demethylation of the methoxylated compound and methyl transfer to the corrinoid protein as well as subsequent methyl transfer to tetrahydrofolate.

In contrast to the well‐studied methoxydotrophic growth of acetogenic bacteria, archaea were not well investigated for their ability of converting methoxylated aromatic compounds. It was only recently, that the thermophilic methanogen *Methermicoccus shengliensis* was shown to be able to use a variety of methoxylated aromatic compounds for growth and produces methane from the methoxy group (Mayumi *et al*., 2016). *M. shengliensis* appears to use a similar *O*‐demethylation system as acetogenic bacteria, composed of the so‐called Mto proteins (Kurth *et al*., submitted): MtoB is the *O*‐demethylase that transfers the methyl group to the corrinoid protein MtoC, MtoD is the reductive activase of MtoC, and MtoA is a methyltransferase that most likely transfers the methyl group to tetrahydromethanopterin (H_4_MPT). Discovery of a methoxydotrophic methanogen together with the prevalence of methoxylated compounds on earth lead us to assume that methoxydotrophic archaea might play a so far underestimated role in the global carbon cycle, especially in the subsurface (Welte, 2016). To further support this hypothesis, we aimed to identify additional methoxydotrophic archaea. A BlastP (NCBI)) analysis of the genes encoding the Mto proteins in *M. shengliensis* (BP07_03250‐60) revealed that the hyperthermophilic archaeon *Archaeoglobus fulgidus* possesses a similar gene cluster, encoding the Mto proteins (AF_0006‐AF_0013). Similar to *Methermicoccus*, *A. fulgidus* (Stetter *et al*., 1987) belongs to the phylum Euryarchaeota and is related to methanogens. The organism grows anaerobically at extremely high temperatures around 80°C and has been described to be present in marine hydrothermal systems (Stetter *et al*., 1987) and oil reservoirs (Beeder *et al*., 1994; Stetter and Huber, 1998). In marine hydrothermal systems, aromatic compounds such as benzenes and phenols have been described as the major organic compounds alongside aliphatic hydrocarbons and carboxylic acids (Konn *et al*., 2009). Concentrations of about 2–4 μg/g soil have been detected in a hydrothermal chimney and sediment (Wang *et al*., 2020). Furthermore, aromatic compounds are a major component (20%–43%) of crude oil (Libes, 2009; Meslé *et al*., 2013). *A. fulgidus* has been described to couple the reduction of sulfate or thiosulfate with the oxidation of many different organic carbon sources such as small organic acids like lactate, pyruvate, formate (Stetter *et al*., 1987; Stetter, 1988) or amino acids like phenylalanine (Parthasarathy *et al*., 2013). The organism also oxidizes fatty acids (C4 to C18) and *n*‐alk‐1‐enes (C12:1 to C21:1) in the presence of thiosulfate as a terminal electron acceptor producing CO_2_ and sulfide (Khelifi *et al*., 2010). Moreover, *A. fulgidus* has been shown to grow on CO and sulfate thereby producing CO_2_, sulfide and acetate (Henstra *et al*., 2007; Hocking *et al*., 2015) and on H_2_ plus CO_2_ with thiosulfate as electron acceptor (Hocking *et al*., 2014). Furthermore, *A. fulgidus* can use perchlorate and nitrate as electron acceptor during hydrocarbon conversion, indicating its very versatile metabolism (Liebensteiner *et al*., 2013, 2014). *A. fulgidus* converts organic acids to CO_2_ via an acetyl‐CoA pathway that involves similar enzymes and cofactors as found in methanogens (Möller‐Zinkhan *et al*., 1989; Möller‐Zinkhan and Thauer, 1990). *A. fulgidus* does not encode methyl‐CoM reductase and thus lacks the potential to produce methane (Klenk *et al*., 1997). In this study, we performed growth experiments, analysed the substrate consumption and product formation and the difference in gene expression of *A. fulgidus* grown on methoxy compounds versus lactate. We demonstrate that *A. fulgidus* can grow on methoxylated aromatic compounds and thereby identify the first non‐methanogenic archaeon capable of methoxydotrophic growth.

## Results

### Growth of *A. fulgidus* on methoxylated aromatic compounds versus lactate

**Fig 1:**
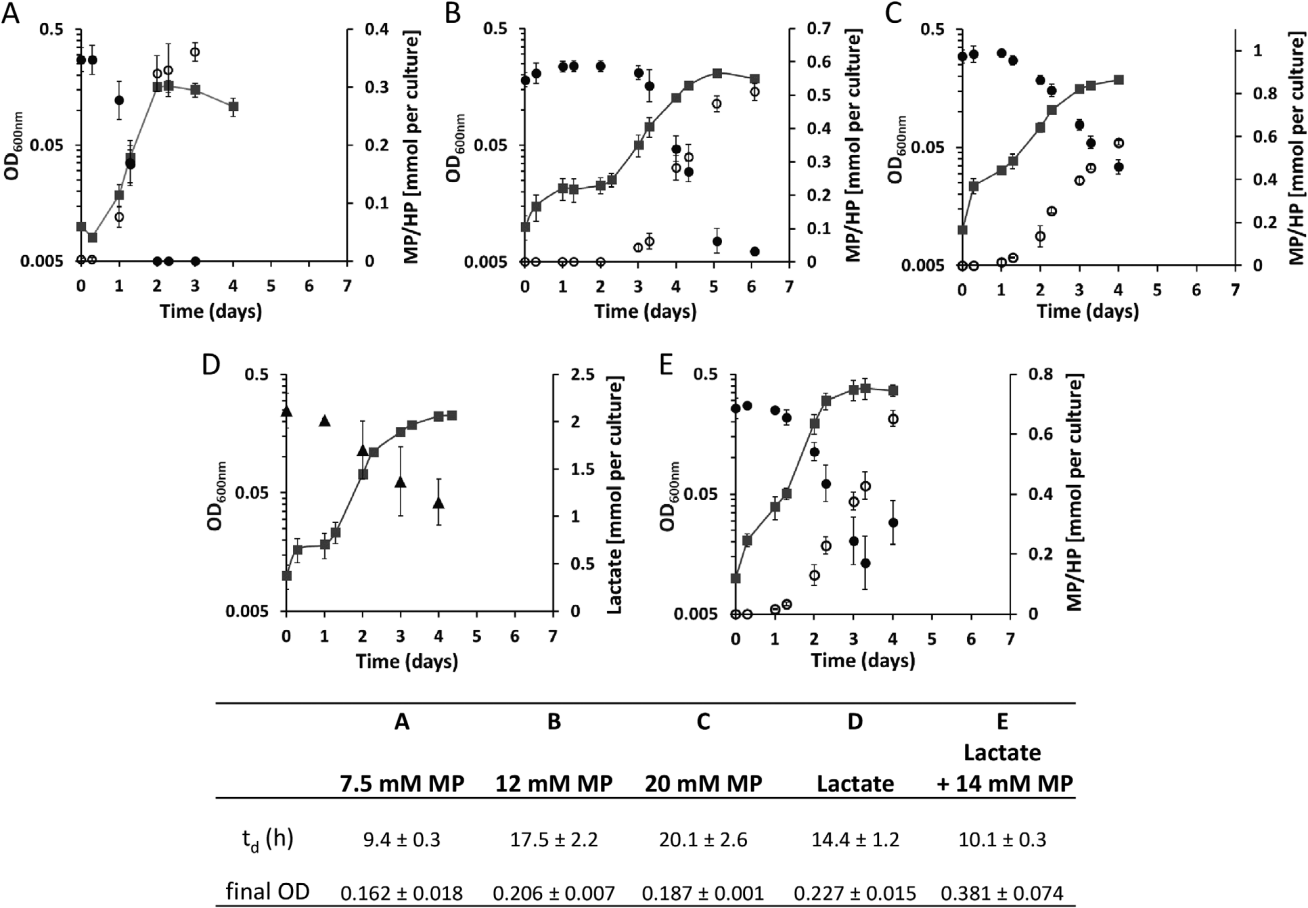
Growth of *A. fulgidus* on 2‐methoxyphenol (MP). *A fulgidus* was grown on either 7.5 mM MP (A), 12 mM MP (B), 20 mM MP (C), 35 mM lactate (D) or 35 mM lactate plus 14 mM MP (E). 2‐methoxyphenol and 2‐hydroxyphenol were determined by HPLC‐UV. Lactate was measured with a colorimetric assay. Grey squares: OD_600nm_, black circles: 2‐methoxyphenol, white circles: 2‐hydroxyphenol, black triangles: lactate. Data are presented as mean +/− standard deviation (*n* = 3).

After a genome analysis, we identified an *O*‐demethylation system in *A. fulgidus*. To experimentally verify if *A. fulgidus* can use this system, we grew the microorganism on 2‐methoxyphenol (MP), and compared this to growth on lactate (Fig. 1). Thus, we could show that *A. fulgidus* is able to grow on MP and converts it to 2‐hydroxyphenol (HP) (Fig. 1A–C). When 7.5 and 12 mM MP were added, MP was completely converted to HP. However, the increase in doubling time (from 9.4 to 20 h) with higher MP concentrations (Fig. 1A–C) points to an inhibiting or toxic effect of high concentrations of methoxylated aromatics (or their products) on the growth of *A. fulgidus*. At 30 mM MP, no growth could be observed. Overall, growth on MP is comparable to growth on lactate (Fig. 1A, B, D), with a similar doubling time (9.4 vs. 14.4 h) of cultures with 7.5 mM MP and a similar final optical density (OD) of cultures with 12 mM MP compared to the lactate containing cultures (see below and Fig. 3 for details). When *A. fulgidus* is cultured in medium containing both lactate and MP, MP improves the growth of the organism resulting in a higher final OD and decreased doubling time (Fig. 1E). Next to 2‐methoxyphenol, growth on methoxylated aromatics such as 2,6‐dimethoxyphenol, methoxyhydroquinone and 2‐methoxybenzoate was observed. No growth could be observed on methanol, indicating that there might be no methanol‐converting enzyme present in *A. fulgidus*.

**Fig 2:**
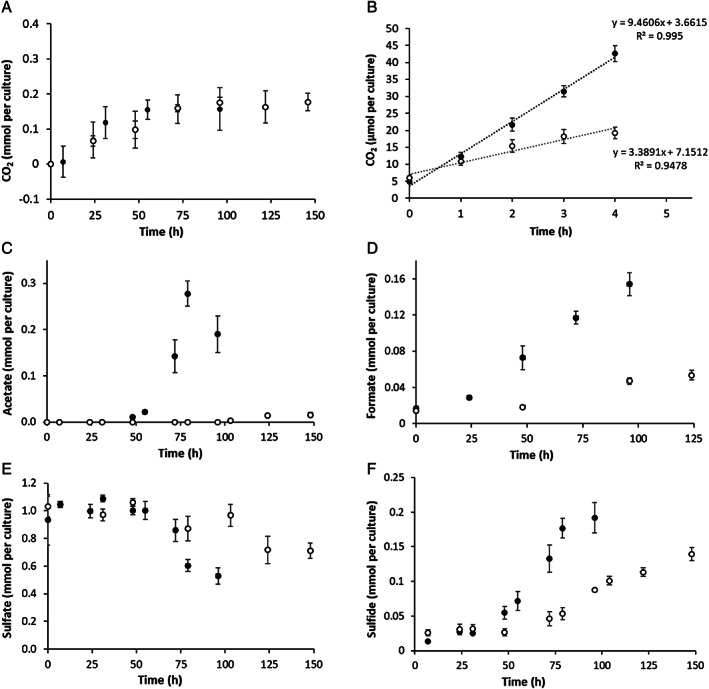
Substrate consumption and product formation during growth of *A. fulgidus* on 12 mM 2‐methoxyphenol (grey circles) versus 34 mM lactate (black circles) with 16 mM sulfate as electron acceptor. Samples for (A), (C), (D), (E) and (F) were taken during the growth experiments shown in Fig. 1B and D. Samples for (B) were taken during a resting cell experiment. CO_2_ and acetate were measured by gas chromatography and formate, sulfate and sulfide with colorimetric assays. Data are presented as mean +/− standard deviation (*n* = 3).

The metabolite production and electron acceptor use of *A. fulgidus* during methoxydotrophic growth were investigated. Therefore CO_2_, acetate, formate, sulfate and sulfide concentrations were measured in experiments with methoxy compounds and lactate (Fig. 2). It appeared that similar amounts of CO_2_ are produced in cultures grown on MP and lactate (Fig. 2A). In a resting cell experiment (Fig. 2B), cells grown on lactate produce (a little) more CO_2_ in the exponential phase than cells grown on methoxyphenol. The reason might be that – during growth – also acetate and formate are produced (Fig. 2C and D), but during the resting cell experiment CO_2_ might be the main product. Another reason could be that cells grown on methoxyphenol were not that metabolically active during the resting cell experiment due to the toxic effect of MP which might be enhanced in the stabilizing buffer used for the experiment compared to medium. The stabilizing buffer contains potassium phosphate, magnesium sulfate, sodium chloride and sucrose, but lacks ammonium, trace elements, yeast extract and CO_2_. The cells are able to metabolize the substrates in this buffer, but are unable to grow. Extrapolation of the resting cell experiment results would lead to CO_2_ production of about 370 μmol CO_2_ per culture produced during growth on MP and 420 μmol during growth on lactate. In medium without added CO_2_ or bicarbonate *A. fulgidus* was only able to grow when lactate was the substrate but not with MP. This indicates that CO_2_ is required for assimilation when MP is the substrate. Next to CO_2_, acetate and formate were major products during growth on lactate (Fig. 2C and D). However, during growth on MP only small amounts of acetate (<20 μM) and formate (<50 μM) were produced (Fig. 2C and D). During both MP and lactate conversion, sulfate is consumed and thereby used as electron acceptor (Fig. 2E). Concomitant sulfide production was also observed (Fig. 2F). Both sulfate consumption and sulfide production were lower in MP grown cells compared to lactate grown cells. In both cases, lower concentrations of sulfide could be measured than sulfate consumed, which might be due to the reactivity of sulfide and/or the use of sulfide for assimilation.

**Fig 3:**
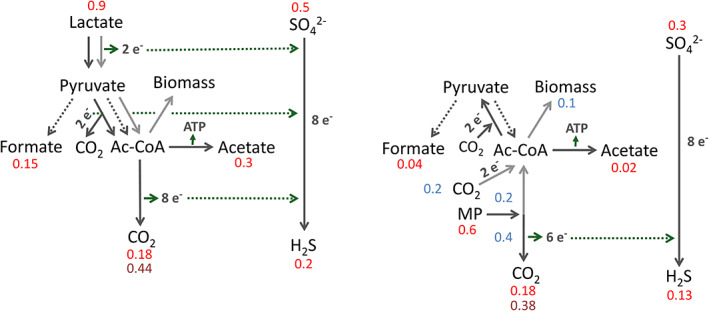
Substrate consumption/product formation during growth of *A. fulgidus* on 12 mM 2‐methoxyphenol versus 34 mM lactate. Values refer to mmol per culture. Red values are based on experimental measurements (see Fig. 2; dark red values refer to resting cell experiment) and blue values are based on estimations (Compensation for the experimentally determined values for metabolites such as CO_2_ and MP). Dark green dotted arrows visualize electrons liberated during carbon metabolism that are consumed during sulfate reduction. Sulfide is most likely underestimated as sulfide reacts with other medium/cell components and is also used for assimilation. [Color figure can be viewed at wileyonlinelibrary.com]

To get a better insight into the metabolism of *A. fulgidus* grown on MP compared to lactate, the concentrations measured for MP, HP, lactate (all shown in Fig. 1) and for CO_2_, acetate, formate, sulfate, sulfide (all shown in Fig. 2) per culture were incorporated into a schematic overview (Fig. 3). During growth on lactate mainly acetate, CO_2_ and formate are produced. Electrons liberated during lactate conversion to pyruvate and acetyl‐CoA as well as during the reductive acetyl‐CoA pathway, resulting in CO_2_ formation, are used for sulfate reduction. During growth on MP, part of the methoxy group is oxidized to CO_2_ resulting in liberation of electrons which can be used for sulfate reduction and the other part is combined with CO_2_ to acetyl‐CoA, from which cell components for assimilation are synthesized. As shown before, only very small amounts of formate or acetate are produced during growth on MP, demonstrating that CO_2_ is the main product. Furthermore, it is possible that amino acids such as glutamate that are present in the yeast extract of the medium might be co‐assimilated, resulting in mixotrophic growth, as it has been described previously that *A. fulgidus* has the genetic potential to degrade a variety of hydrocarbons and organic acids (Klenk *et al*., 1997).

A metabolite analysis of *A. fulgidus* cells grown under different conditions (Supporting Information Fig. S1) revealed that *A. fulgidus* cells contained less pyruvate, succinate, citrate, fumarate when grown on MP compared to growth on lactate, which can be partly explained by the growth of *A. fulgidus* on MP and its conversion via the reductive acetyl‐CoA pathway instead of growth on organic acids. Furthermore, 2‐oxoglutarate was only detected in lactate‐grown cultures. In contrast, glutamate was detected in all cultures in comparable amounts.

### Transcriptomic analysis

**Fig 4:**
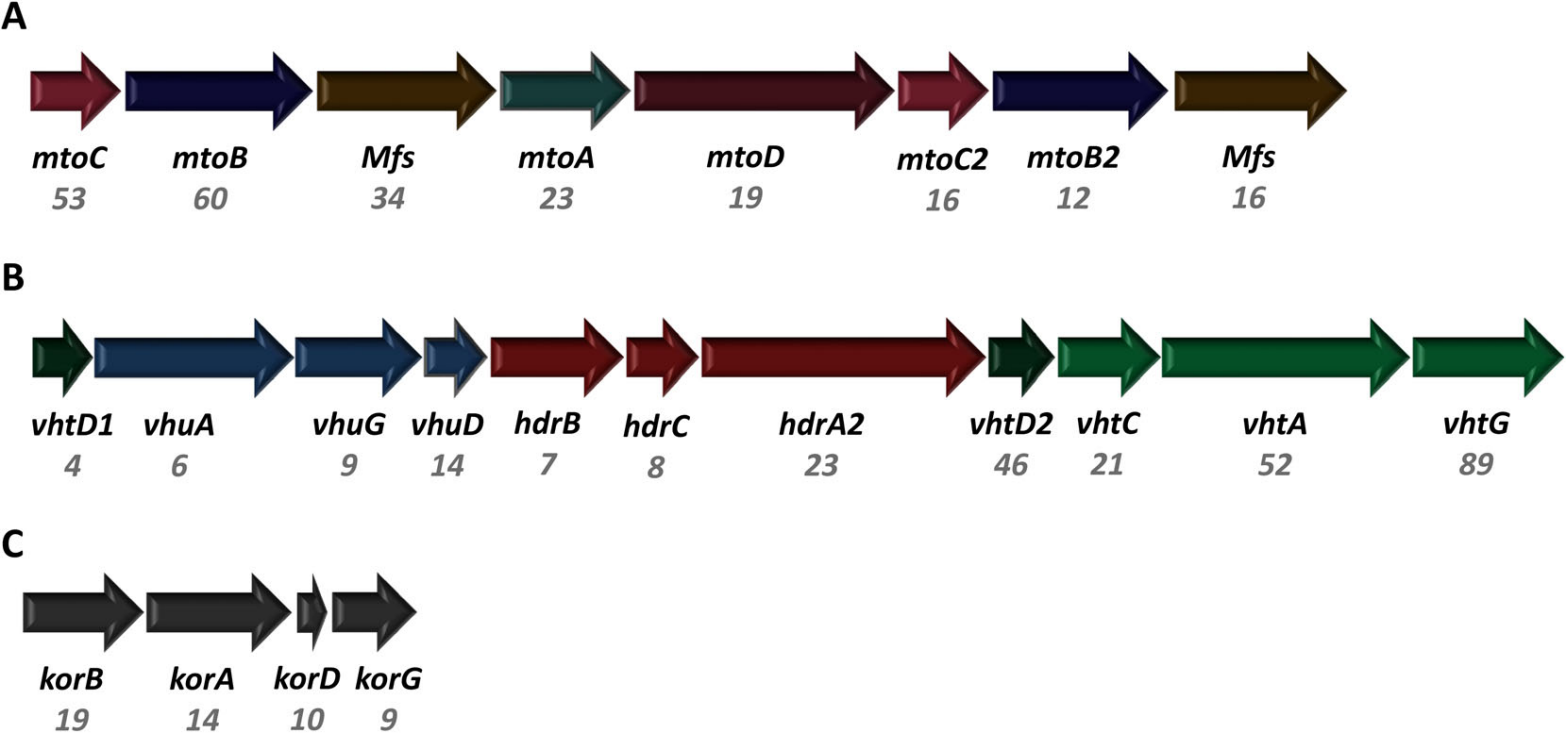
Gene clusters upregulated under growth on MP versus lactate with corresponding fold change values. The gene clusters depicted here encode (A) proteins involved in demethoxylation (AF_0006–13), (B) hydrogenases (AF_1371‐81) and (C) the 2‐oxoglutarate/2‐oxoacid ferredoxin oxidoreductase KorABDG (AF_0468‐71). MtoC: Cobalamin binding protein, MtoD: corrinoid activator protein, MtoB: *O*‐demethylase, MtoA: methyl transferase, MFS: major facilitator superfamily transporter, HdrABC: soluble heterodisulfide reductase, Vht and Vhu: F_420_‐nonreducing hydrogenase, KorABDG: 2‐oxoglutarate/2‐oxoacid ferredoxin oxidoreductase. The difference in gene expression of those genes after growth on MP or lactate is shown in Supporting Information Table S2. Fold change values are depicted in grey. [Color figure can be viewed at wileyonlinelibrary.com]

**Fig 5:**
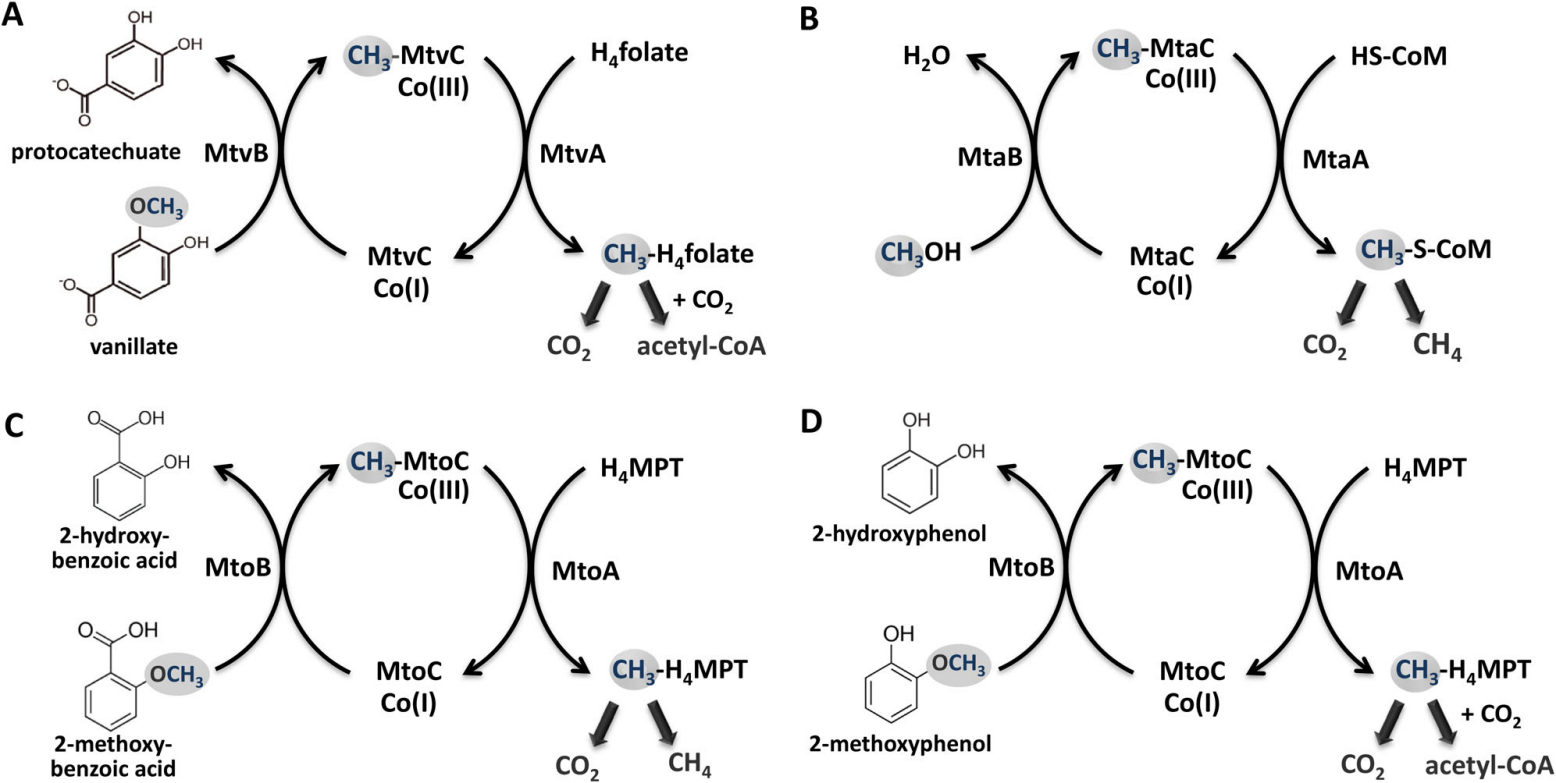
Demethylation and demethoxylation pathways of methoxydotrophic archaea in comparison to methylotrophic methanogens and acetogenic bacteria. (A) Demethoxylation pathway as described for the acetogenic bacterium *Moorella thermoacetica*, modified from Pierce *et al*. (Pierce *et al*., 2008). (B) Demethylation pathway in methanogenic archaea when using methanol as substrate. (C) Demethoxylation pathway of the methanogen *M. shengliensis* (Mayumi *et al*., 2016; Kurth *et al*., submitted). (D) Tentative demethoxylation pathway of *A. fulgidus*. Genomic and transcriptomic analysis revealed cobalamin binding protein MtoC (AF_0006) and its activator MtoD (AF_0010), O‐demethylase MtoB (AF_0007) and methyl transferase MtoA (AF_0009) to be essential for growth of *A. fulgidus* on methoxylated aromatic compounds. CoM: coenzyme M, H_4_folate: tetrahydrofolate, CO(III): cobalamin binding protein, H_4_MPT: tetrahydromethanopterin. [Color figure can be viewed at wileyonlinelibrary.com]

After analysing which products were formed during growth on MP and lactate, we also wanted to investigate how the gene expression differs under the two growth conditions and which genes are important for methoxydotrophic growth (Supporting Information Tables S1 and S2). The three most upregulated gene clusters under methoxydotrophic growth compared to growth on lactate are shown in Fig. 4. The first gene cluster (Fig. 4A) contains the genes that were identified for *M. shengliensis* to be important for growth on methoxy compounds (Kurth *et al*., submitted). This gene cluster encodes the *O*‐demethylase MtoB, which is responsible for *O*‐demethylation of the methoxy compound, the cobalamin binding protein MtoC that accepts the methyl group from MtoB, the reductive activase of MtoC named MtoD, the methyl transferase MtoA which most likely transfers the methyl group from MtoC to tetrahydomethanopterin (H_4_MPT) and major facilitator superfamily transporters that might be involved in import of the methoxylated aromatic compounds and export of the hydroxylated derivatives. The role of these enzymes is depicted in Fig. 5.

**Fig 6:**
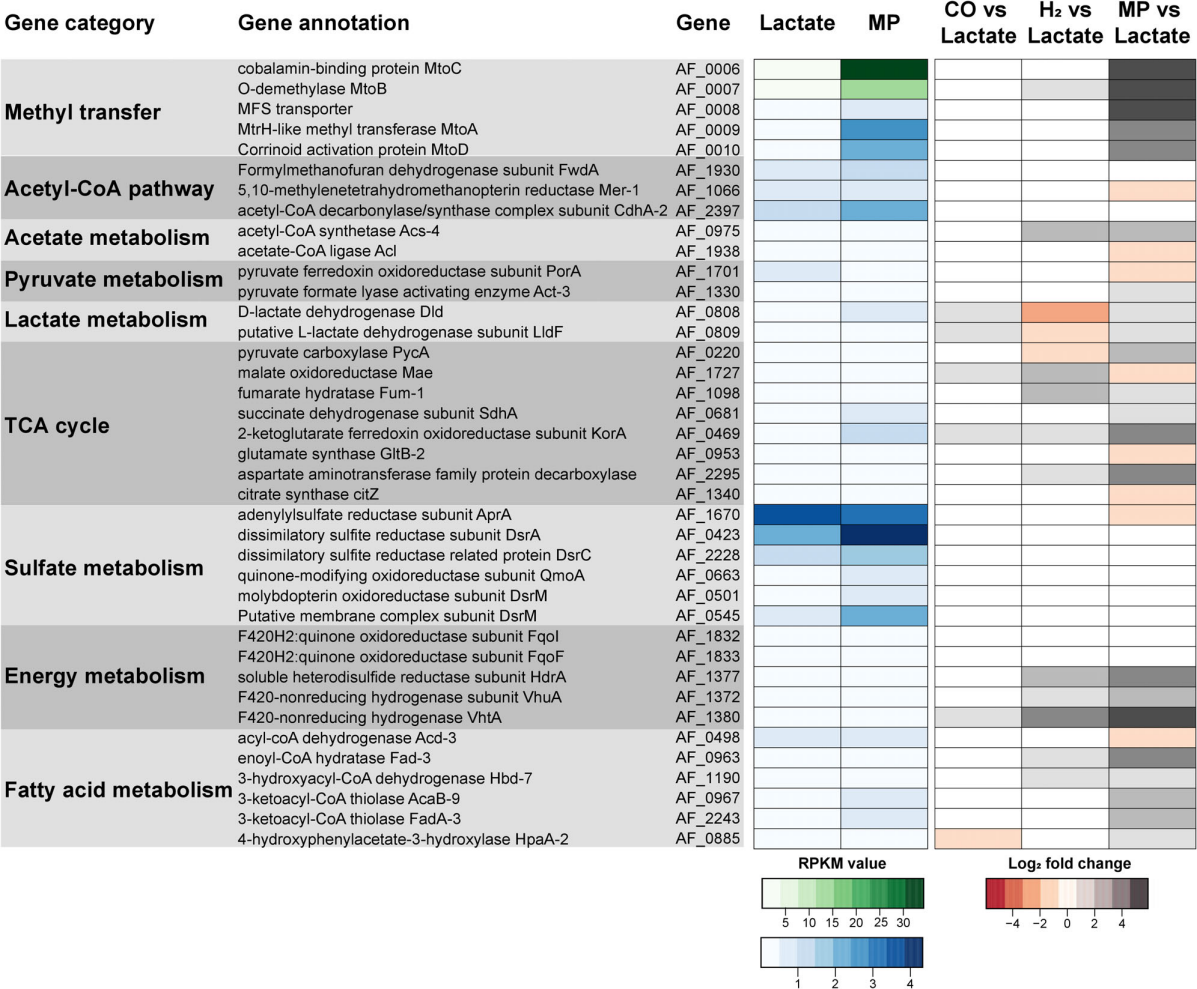
Comparison of gene expression during growth of *A. fulgidus* on lactate to growth on 2‐methoxyphenol, H_2_/CO_2_ or CO. RPKM (reads per kilobase transcript per million mapped reads) values were normalized to the average S3 ribosomal protein (gene AF_1919) RPKM under growth on lactate or 2‐methoxyphenol (MP). RPKM values for AF_0006 and AF_0007 are depicted with a different scale (green shades) than the other genes (blue shades). Log_2_fold change values are shown for MP versus lactate (this study), H_2_/CO_2_ versus lactate (Hocking *et al*., 2014) and CO versus lactate (Hocking *et al*., 2015) (red/grey shades). Non‐normalized RPKM values and log_2_fold change values can also be found in Supporting Information Tables S1–S3. [Color figure can be viewed at wileyonlinelibrary.com]

Another highly upregulated gene cluster encodes for two F_420_‐non‐reducing hydrogenases, Vht and Vhu, and for a heterodisulfide reductase (Hdr) complex. The cytoplasmic Vhu hydrogenase (also referred to as Mvh hydrogenase) is assumed to be associated with the Hdr complex (Hocking *et al*., 2014). The Vht hydrogenase contains a membrane subunit and is most likely located in the pseudoperiplasm. As no hydrogen gas was added to the cultures and no production of hydrogen could be detected, there is most likely an internal hydrogen cycling occurring in the cells. H_2_ might be produced by the Hdr/Vhu complex and reoxidized by the periplasmic Vht hydrogenase, contributing to the proton gradient and reducing menaquinone. The third gene cluster that is highly overexpressed under MP growth encodes the 2‐oxoglutarate/2‐oxoacid ferredoxin oxidoreductase KorABDG. This enzyme can be part of the tricarboxylic acid (TCA) cycle and catalyses the reversible conversion of 2‐oxoglutarate to succinyl‐CoA and reduction of ferredoxin, therefore potentially playing a role in regenerating/providing reducing equivalents. The 2‐oxoglutarate might derive from glutamate which is present in the yeast extract provided to the medium. Next to those three gene clusters also genes encoding for proteins involved in lipid metabolism, more precisely fatty acid activation and β‐oxidation, are highly upregulated. Those highly upregulated genes encode for proteins such as the long‐chain‐fatty‐acid‐CoA ligase FadD, the acyl‐CoA dehydrogenase Acd, the enoyl‐CoA hydratase Fad, the 3‐hydroxyacyl‐CoA dehydrogenase Hbd, the 3‐ketoacyl‐CoA thiolase AcaB and FadA, the medium‐chain acyl‐CoA ligase AlkK and the 4‐hydroxybutyrate CoA transferase Cat2 (Fig. 6, Supporting Information Tables S1 and S2). Other genes that are strongly induced under MP growth are genes encoding branched‐chain amino acid ABC transporter subunits (Supporting Information Table S2) involved in amino‐acid uptake, genes encoding acyl‐CoA synthetase Acs, which might play a role in acetate activation or fatty acid activation, as well as genes encoding putative acyl‐CoA transferase/formyl‐CoA transferases. Similar observations were made in a transcriptomic study with *A. fulgidus* cell grown on H_2_/CO_2_ versus lactate (Hocking *et al*., 2014) and might therefore be associated with the growth of *A. fulgidus* on substrates other than organic acids and putatively the co‐assimilation of lipids, amino acids and other organic compounds, resulting in mixotrophic growth.

Genes that are downregulated during growth on MP (Supporting Information Table S3) encode proteins involved in ammonia uptake (Amt) and glutamine metabolism/nitrogen regulation (GlnA, GlnB), ferrous iron transport (FeoB), phosphate transport (PstSABC) as well as pyruvate metabolism (PorABDG). Ammonia and glutamine metabolism might be downregulated because of co‐assimilation of amino acids such as glutamate, which would match with the upregulation of *korABDG*. Genes encoding the pyruvate ferredoxin oxidoreductase PorABDG are most likely downregulated as less pyruvate is produced due to the lack of lactate conversion. The downregulation of genes involved in ferrous iron and phosphate uptake might correlate with a decreased production of iron–sulfur and polyphosphate bodies that have been described for *A. fulgidus* cells (Toso *et al*., 2016). The iron–sulfur bodies store iron, sulfur plus copper and the polyphosphate bodies contain phosphorus plus magnesium, calcium, and aluminium. Those iron–sulfur and polyphosphate bodies are assumed to be involved in energy storage and/or metal sequestration/detoxification.

**Fig 7:**
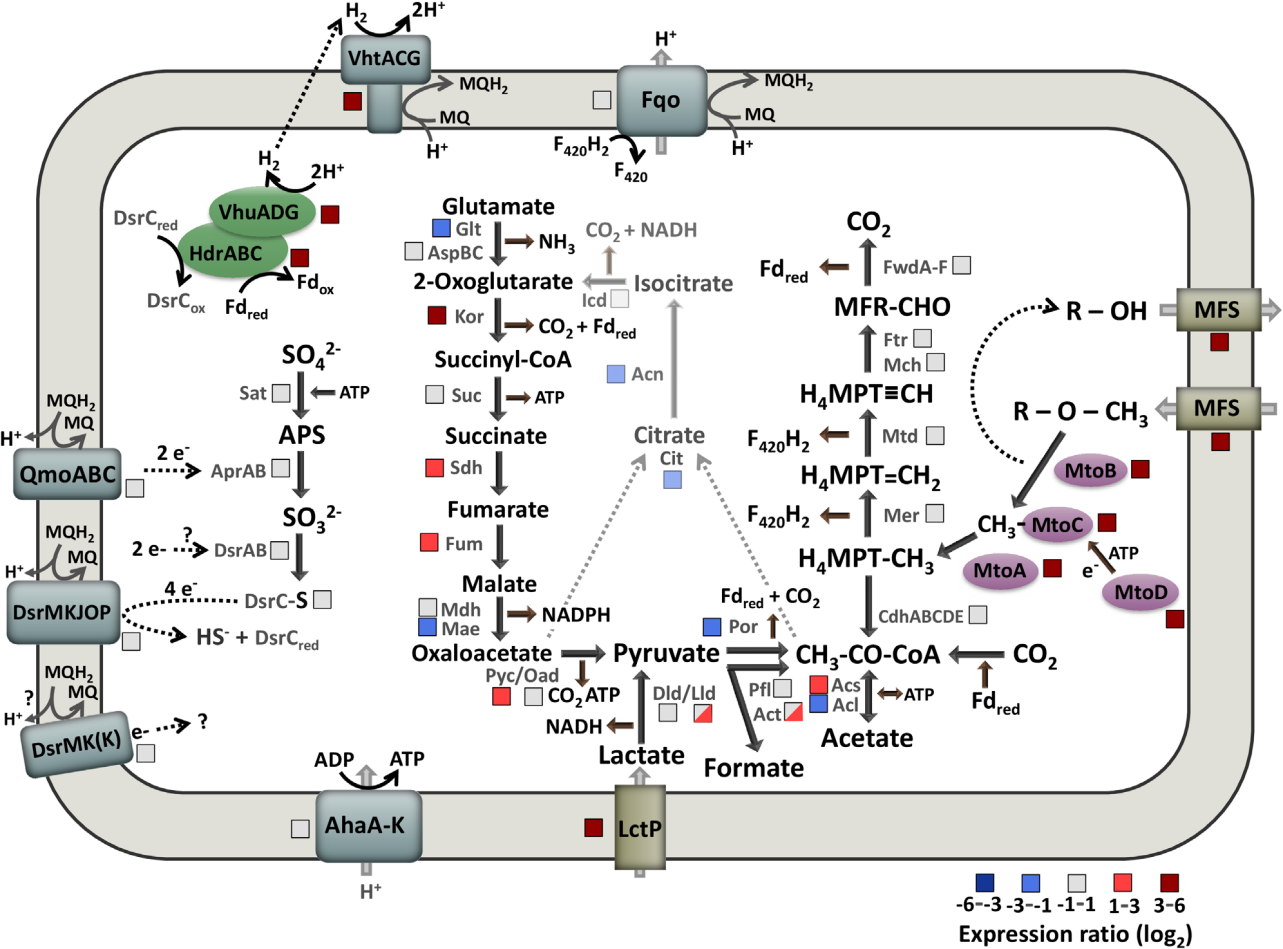
Tentative pathway for methoxydotrophic growth in *A. fulgidus*. RPKM and log_2_ fold change values for all proteins shown in this figure are included in Supporting Information Table S1. Genes encoding the following enzymes can be found in *A. fulgidus* VC‐16: Formylmethanofuran dehydrogenase FwdA‐F (AF_0177, AF_1644, AF_1649‐51, AF_1928‐31), formylmethanofuran‐tetrahydromethanopterin formyl‐transferase Ftr (AF_2073 & AF_2207), methenyl‐tetrahydromethanopterin cyclohydrolase Mch (AF_1935), methylenetetrahydromethanopterin dehydrogenase Mtd (AF_0714), 5,10‐methylenetetrahydromethanopterin reductase Mer (AF_1066 & AF_1196), acetyl‐CoA decarbonylase/synthase CODH/ACS complex CdhABCDE (AF_0376/7/9, AF_1100/1, AF_2397/8), acetyl‐CoA synthetase Acs1‐5 (AF_0197, AF_0366, AF_0677, AF_0975, AF_0976) and acetate‐CoA ligase Acl (AF_1211 & AF_1938), pyruvate ferredoxin oxidoreductase PorABDG (AF_1699–1702), pyruvate formate lyase PflCD (AF_1449/50), PflX (AF_1961) and Act‐1‐4 (AF_0117, AF_0918, AF_1330, AF_2278), D‐lactate dehydrogenase Dld (AF_0394 & AF_0808), L‐lactate dehydrogenase LldD (AF_0807) and LldEFG (AF_0809‐AF_0811), lactate permease lctP (AF_0806), pyruvate carboxylase PycA (AF_0220) and oxaloacetate decarboxylase Oad (AF_1252), malate dehydrogenase MdhA (AF_0855) and malate oxidoreductase Mae (AF_1727), fumarate hydratase Fum‐1/2 (AF_1098/9), succinate dehydrogenase SdhA‐D (AF_0681‐4), succinyl‐CoA synthetase SucCD (AF_1539/40 & AF_2185/6), 2‐oxoglutarate/2‐oxoacid ferredoxin oxidoreductase KorABDG (AF_0468‐71), glutamate synthase GltB1‐3 (AF_0952‐4), aspartate aminotransferase AspBC (AF_0409, AF_1623, AF_2129, AF_2366, AF_1417), isocitrate dehydrogenase Icd (AF_0647), aconitase Acn (AF_1963), citrate synthase CitZ (AF_1340), sulfate adenylyltransferase Sat (AF_1667), adenylylsulfate reductase AprAB (AF_1669‐70), dissimilatory sulfite reductase DsrAB (AF_0423‐4), DsrC (AF_2228), quinone‐modifying oxidoreductase QmoABC (AF_0661‐3), molybdopterin oxidoreductase DsrMKJOP (AF_0499‐503), DsrMK(K) (AF_0543‐5), F_420_H_2_:quinone oxidoreductase FqoJKMLNABCDHIF (AF_1823‐33), soluble heterodisulfide reductase HdrABC (AF_1375‐7), F_420_‐non‐reducing hydrogenase VhuADG (AF_1372‐4), F_420_‐nonreducing hydrogenase VhtACDG (AF_1378‐81), ATP synthase AtpA‐K (AF_1158‐68), cobalamin binding protein MtoC (AF_0006) and its activator MtoD (AF_0010), O‐demethylase MtoB (AF_0007) and methyl transferase MtoA (AF_0009), MFS transporters (AF_0008 & AF_0013). H_4_MPT: tetrahydromethanopterin, MQH_2_: reduced menaquinone (MQ), MFR: methanofuran, Fd: ferredoxin, F_420_H_2_: reduced coenzyme F_420_, MFS: major facilitator superfamily transporter. Expression ratio shown for *A. fulgidus* grown on MP versus lactate. [Color figure can be viewed at wileyonlinelibrary.com]

Proteins involved in the metabolism of methoxylated aromatics, acetyl‐CoA/Wood‐Ljungdahl pathway, lactate metabolism, TCA cycle, sulfate reduction, energy metabolism and regeneration of reducing equivalents are shown in Fig. 7 (for details see also Fig. 6 and Supporting Information Table S1) with the respective transcription profile for growth on MP versus lactate. There is no significant change in transcription regarding genes encoding proteins involved in sulfate reduction and acetyl‐CoA pathway. This matches with the observation that sulfate serves as electron acceptor and CO_2_ is produced under both growth conditions. Regarding energy metabolism and regeneration of reducing equivalents, only genes encoding for the Hdr complex and the two hydrogenases (Vht and Vhu) are upregulated on MP. In view of the TCA cycle, some of the genes encoding proteins involved in the stepwise conversion from 2‐oxoglutarate to pyruvate are upregulated (KorABDG, SdhABCD, Fum, Pyc), whereas genes encoding proteins involved in citrate and isocitrate formation are downregulated (Cit, Acn). Those observations are discussed below.

## Discussion

### Methoxydotrophic growth – *O*‐demethylation of methoxylated aromatic compounds

In this study, we demonstrate that *A. fulgidus* can grow on methoxylated aromatic compounds such as 2‐methoxyphenol. Similar to some acetogenic bacteria, e.g. *Sporomusa termitida* (Breznak *et al*., 1988), *Clostridium thermoaceticum* (Wu *et al*., 1988) and *Acetobacterium woodii* (Bache and Pfennig, 1981), as well as the methanogenic archaeon *M. shengliensis* (Kurth *et al*., submitted; Mayumi *et al*., 2016), *A. fulgidus* converts methoxylated aromatic compounds to their hydroxylated derivatives (Fig. 1). Surprisingly, both the archaea *M. shengliensis* and *A. fulgidus* use an *O*‐demethylation system that is similar to that of acetogenic bacteria (Fig. 5 and Kurth *et al*., submitted). The enzymes that are part of this system are encoded in a gene cluster that is highly induced under methoxydotrophic conditions (Fig. 4A). These MtoABCD proteins facilitate *O*‐demethylation of the methoxy compound and methyl transfer via a corrinoid protein to most probably tetrahydromethanopterin (H_4_MPT) (Kurth *et al*., submitted). The acetyl‐CoA pathway of *A. fulgidus* has been shown to rather involve tetrahydromethanopterin than tetrahydrofolate as C1‐carrier (Möller‐Zinkhan *et al*., 1989) which differs to acetogenic bacteria but strengthens the hypothesis that MtoA transfers the methyl group to H_4_MPT in *A. fulgidus*. The produced methyl‐H_4_MPT can then be oxidized to CO_2_ via the reductive acetyl‐CoA pathway, generating reducing equivalents which provide electrons for sulfate reduction directly via ferredoxin or indirectly via reduced menaquinone (Fig. 7). We could further show that growth characteristics for MP as substrate were comparable to those for lactate (Fig. 1). However, MP concentrations of 12 mM and higher had an inhibiting effect on growth, which might be due to the toxic effect of aromatic hydrocarbons or their products. At lower concentrations, however, methoxy compounds improved the growth of *A. fulgidus* in the presence of lactate. As *A. fulgidus* thrives in environments such as hydrothermal marine systems (Stetter *et al*., 1987) and oil reservoirs (Beeder *et al*., 1994; Stetter and Huber, 1998) where aromatic compounds are present (Konn *et al*., 2009; Libes, 2009; Meslé *et al*., 2013) the ability to use methoxylated aromatics for growth might lead to a growth advantage over organisms that are incapable of methoxydotrophic growth. Furthermore, the use of methoxylated aromatics as co‐substrate to organic acids such as acetate or lactate, amino acids, fatty acids or sugars might be more prevalent than so far assumed in marine environments and subsurface sediments as methoxy compounds are quite abundant on earth.

**Fig 8:**
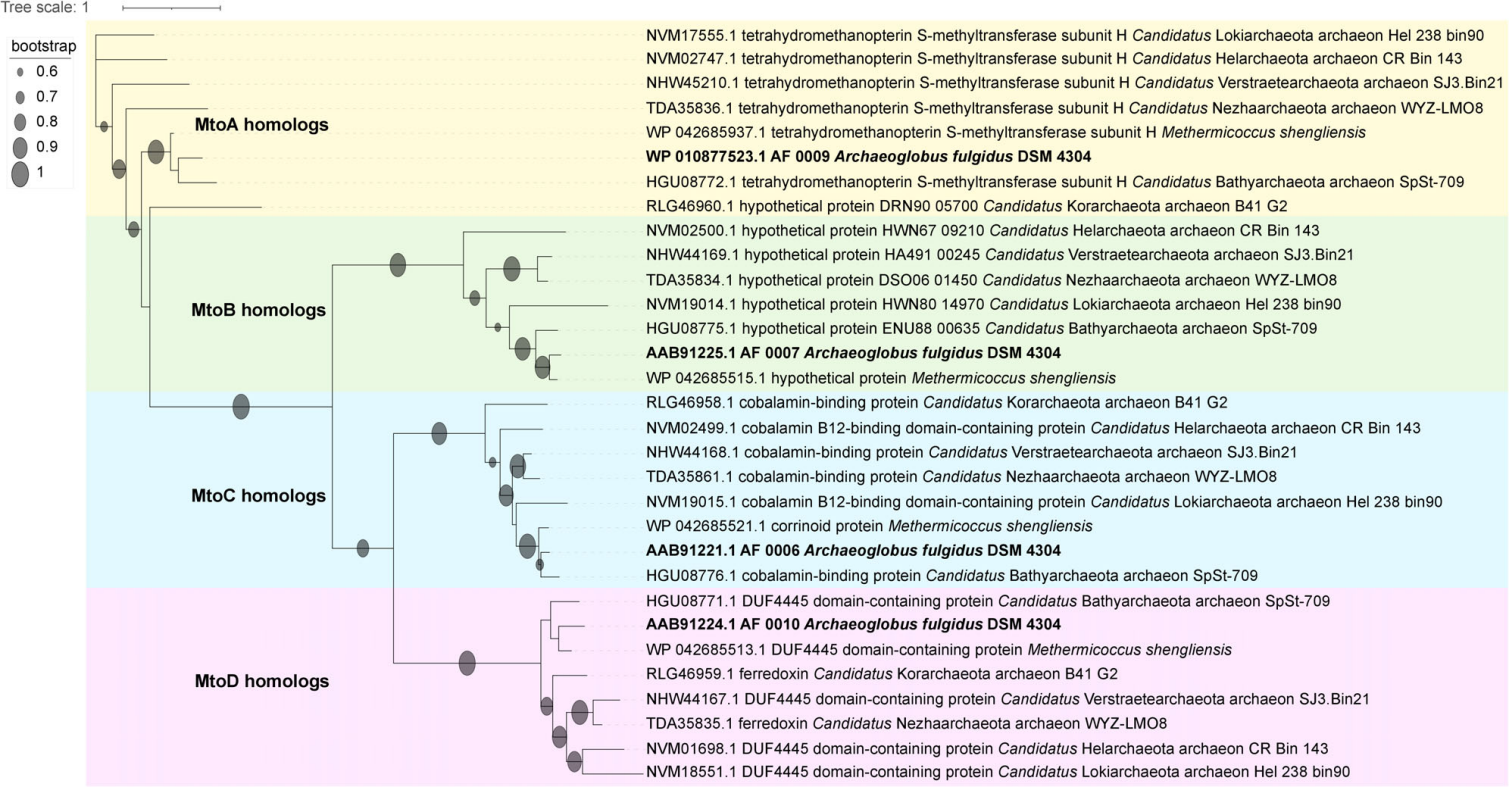
Phylogenetic tree of Mto proteins from *A. fulgidus* and putative homologues from other archaea. The tree was generated with FastTree using the Jones‐Taylor‐Thorton maximum likelihood model. Sequence accession numbers from NCBI are provided in the tree. [Color figure can be viewed at wileyonlinelibrary.com]

Bioinformatic analysis revealed that also other archaea that thrive in hydrothermal deep‐sea sediments such as members of the phyla Bathyarchaeota, Lokiarchaeota, Korarchaeota, Helarchaeota, Verstraetearchaeota and Nezhaarchaeota contain *mtoABC* homologues in their genomes (Fig. 8). The latter three organisms are assumed to be involved in methane and/or alkane metabolism (Hua *et al*., 2019; Seitz *et al*., 2019; Wang *et al*., 2019). Bathyarchaeota are very abundant in marine subsurface sediments and have the potential for acetogenesis and for the fermentative utilization of a variety of organic substrates (He *et al*., 2016). Furthermore, they have been described to be able to grow on lignin (Yu *et al*., 2018). Also for Lokiarchaeota, it is assumed that they have the potential to degrade lignin besides other substrates, such as humic acids, lactate, aromatic compounds and proteins (Yin *et al*., 2020). The presence of *mto* homologues in those archaea therefore might suggest that they are able to grow on MACs.

### Metabolism – products formed and electron acceptor used during methoxydotrophic growth

We observed that next to 2‐hydroxyphenol, CO_2_ is the main carbon compound produced by *A. fulgidus* during growth on MP, whereas substantial amounts of acetate and formate are produced besides CO_2_ during growth on lactate (Fig. 2). During methoxydotrophic growth, the methoxy/methyl group ends up as methyl‐H_4_MPT in the acetyl‐CoA pathway and is then either oxidized to CO_2_ resulting in generation of reducing equivalents or combined with CO_2_ to form acetyl‐CoA which is required for assimilation (Fig. 3). When lactate is used as carbon and energy source, reducing equivalents are generated during oxidation of lactate to acetyl‐CoA via pyruvate, but also during oxidation of acetyl‐CoA to CO_2_ in the reductive acetyl‐CoA pathway. Moreover, ATP can directly be generated from the conversion of acetyl‐CoA to acetate. Formate and CO_2_ are side products from the oxidation of pyruvate to acetyl‐CoA. The produced acetyl‐CoA can further also be used for assimilation. Production of acetate, formate, and CO_2_ from lactate has already been shown previously and the stoichiometry of the catabolic reaction seems to strongly dependent on the sulfate concentration (Habicht *et al*., 2005). In our study, sulfate was reduced to sulfide under both growth conditions and therefore serves as electron acceptor.

### Energy metabolism and regeneration of reducing equivalents

Regarding energy metabolism and regeneration of reducing equivalents, mainly genes encoding for the Hdr complex and the two hydrogenases (Vht and Vhu) are upregulated (Fig. 7 and Supporting Information Table S1). This might be due to an increased concentration of reduced ferredoxin (Fd_red_) in the cells which most likely result from increased production of Fd_red_ in the reductive acetyl‐CoA pathway (CO_2_ is the main carbon compound produced during growth on MP), the putative increased production of Fd_red_ by 2‐oxoglutarate ferredoxin oxidoreductase KorABDG (upregulation of *korABDG* genes) and a decrease in sulfate reduction (less sulfate is reduced during growth on MP) which might involve Fd_red_ as electron donor as Qmo and DsrAB have Fd binding sites (Hocking *et al*., 2014). Although less Fd_red_ is assumed to be produced by pyruvate ferredoxin oxidoreductase PorABDG (downregulation of *porABDG* genes), there might still be an increased concentration of Fd_red_ in cells grown on MP compared to cells grown on lactate. Fd_red_ is the most likely electron donor for the Hdr/Vhu complex, as cultures are not grown on H_2_. Reoxidation of Fd_red_ by the Hdr/Vhu complex putatively involves oxidation of DsrC (two SH‐groups) and reduction of H^+^ to H_2_, which could then be oxidized by the pseudoperiplasmic Vht hydrogenase contributing to the proton motive force. The internal hydrogen cycling under methoxydorophic growth conditions is supported by the observation that there was no net production or consumption of hydrogen in the cultures. For the sulfate‐reducing bacterium *Desulfovibrio gigas*, it has been described that hydrogen cycling takes place in lactate‐grown cultures and led to the assumption that hydrogen cycling with vectorial electron transfer might be a general mechanism for energy coupling in sulfate‐reducing bacteria (Odom and Peck, 1981). However, in *A. fulgidus* the hydrogenases are hardly induced in lactate grown cultures (this study and Hocking *et al*., 2014; see also Fig. 6) indicating that hydrogen cycling only plays a role when the organism grows on substrates other than organic acids and H_2_. Under growth on H_2_/CO_2_ and partly also under growth on CO compared to growth on lactate the genes encoding the aforementioned hydrogenases and Hdr proteins are also upregulated (Fig. 6 and Hocking *et al*., 2014, 2015) indicating that they play a general role in energy metabolism and regeneration of reducing equivalents during autotrophic or mixotrophic growth of *A. fulgidus*. Internal hydrogen cycling has also been observed in acetogens such as *Acetobacterium woodii* (Wiechmann *et al*., 2020).

Regarding the TCA cycle, some of the genes encoding proteins involved in the stepwise conversion from 2‐oxoglutarate to pyruvate are upregulated (KorABDG, SdhABCD, Fum, Pyc), whereas genes encoding proteins involved in citrate and isocitrate formation are downregulated (Cit, Acn) (for comparison Fig. 7 and Supporting Information Table S1). This might be due to the upregulation of genes encoding the 2‐oxoglutarate ferredoxin oxidoreductase KorABDG, which might play a role in Fd_red_ production and therefore providing/regenerating reducing equivalents. The metabolite analysis of *A. fulgidus* cells (Supporting Information Fig. S1) revealed that *A. fulgidus* cells contained less of some TCA cycle metabolites such as succinate, or fumarate when grown on MP compared to growth on lactate. Next to the lack of lactate and pyruvate also an upregulation of genes encoding for TCA cycle proteins might explain this finding and fits to the transcriptomic results mentioned before. The absence of 2‐oxoglutarate in MP‐grown cells (Supporting Information Fig. S1) is in agreement with the upregulation of the *korABDG* genes and further indicates that KorABDG indeed converts 2‐oxoglutarate to succinyl‐CoA resulting in Fd_red_ production and does not primarily catalyse the reverse reaction. Furthermore, less Fd_red_ is produced by PorABDG due to the lack of pyruvate providing compounds such as lactate which was evidenced in this study by downregulation of the *porABDG* genes and the metabolite analysis (Supporting Information Table S1 and Fig. S1). KorABDG might compensate for that by providing Fd_red_. However, the source of 2‐oxoglutarate could not be detected yet. We assume that glutamate or other amino acids might be converted to 2‐oxoglutarate as amino acids are present in the yeast extract provided to the medium. However, putative glutamate converting enzymes such as the aspartate aminotransferase AspBC are not upregulated under methoxydotrophic growth. However, a quarter of the *A. fulgidus* genome encodes functionally uncharacterized proteins (Klenk *et al*., 1997) so we might have overlooked a hitherto undescribed glutamate converting enzyme. A gene encoding an aspartate aminotransferase family protein/glutamate decarboxylase is highly upregulated on MP (AF_2295) which could have a role in glutamate conversion. It might also be possible that glutamate is transformed into γ‐aminobutyrate and that KorABDG plays a role in the GABA shunt. For *Mycobacterium tuberculosis* it has been shown that α‐ketoglutarate dehydrogenase defends the bacterium against glutamate anaplerosis and oxidative stress caused by reactive nitrogen species (Maksymiuk *et al*., 2015) indicating that the genes encoding enzyme might be induced during methoxydotrophic growth of *A. fulgidus* due to stress response. Furthermore, this enzyme seems to have a more important role during growth on MACs than growth on H_2_/CO_2_ or CO as genes encoding this enzyme are not upregulated under those conditions (Hocking *et al*., 2014, 2015) (see also Fig. 6) demonstrating that its upregulation might correlate with a stress response caused by methoxydotrophic growth. Also, the genes encoding the cytochrome *bd* ubiquinol oxidase subunits AF_2296 and AF_2297 are strongly upregulated under methoxydotrophic growth (Supporting Information Table S2) which might be due to an oxidative stress responds.

### Lipid and amino acid metabolism

*A. fulgidus* possesses 57 genes encoding β‐oxidation enzymes indicating that the organism is able to degrade a variety of hydrocarbons and organic acids (Klenk *et al*., 1997). In this study, we observed that a vast number of genes encoding for proteins involved in lipid metabolism, amino acid uptake, and acetate/fatty acid activation are upregulated in *A. fulgidus* cells grown on methoxylated aromatics versus lactate. In a transcriptomic study with *A. fulgidus* cells grown on H_2_/CO_2_ versus lactate, similar observations were made (Hocking *et al*., 2014; see also Fig. 6). The upregulation of those genes might enable the organism to co‐assimilate lipids, amino acids and other organic compounds, resulting in mixotrophic growth. Furthermore, some of the upregulated genes (AF_0333, AF_0885, AF_1027) might encode for enzymes that are part of a 3‐hydroxypropionate/4‐hydroxybutyrate (3HP/4HB) cycle (Hocking *et al*., 2014) identified in *Metallosphaera sedula* (Berg *et al*., 2007) and might represent a secondary carbon fixation pathway in *A. fulgidus*. However, in a study with *Archaeoglobus lithotrophicus*, no enzyme activities associated with the dicarboxylate/hydroxybutyrate or the hydroxypropionate/hydroxybutyrate cycles were detected (Estelmann *et al*., 2011).

## Conclusion

In summary, we describe the first non‐methanogenic methoxydotrophic archaeon. Similar to the so far first and only described methoxydotrophic archaeon *M. shengliensis*, which uses methoxylated aromatics for methane production, a bacterial‐like demethoxylation system is used in *A. fulgidus*. In contrast to *M. shengliensis*, *A. fulgidus* converts the methoxy group primarily to CO_2_ and not to CH_4_ by using the reductive acetyl‐CoA pathway, hereby representing a novel type of archaeal metabolism. We showed that *A. fulgidus* can grow on a variety of methoxylated aromatic compounds with a comparable growth rate and growth yield as on lactate and we demonstrated that those compounds improve the growth when lactate is present as a substrate. Other archaea that thrive in hydrothermal deep‐sea sediments such as Bathyarchaeota, Lokiarchaeota, Verstraetearchaeota, Korarchaeota, Helarchaeota and Nezhaarchaeota also appear to have the genetic potential for methoxydotrophic growth. As methoxylated aromatic compounds are quite abundant on earth, especially in the subsurface, we hypothesize that methoxylated aromatic compounds serve as a growth substrate, either on their own or as co‐substrate, for more microorganisms than previously assumed and that methoxydotrophic growth might play an underestimated role in the global carbon cycle.

## Experimental procedures

### Cultivation of *Archaeoglobus fulgidus*

*Archaeoglobus fulgidus* VC‐16 (DSM 4304) was cultivated in anoxic, carbonate buffered medium (50 ml medium in 120 ml glass bottles) under an atmosphere of N_2_:CO_2_ 80:20 (1 atm), at pH 6.8, similar as described by Hocking *et al*. (2014). The composition of the media was as follows: 0.32 g/l KCl, 4.0 g/l MgCl_2_ × 6 H_2_O, 0.25 g/l NH_4_Cl, 0.14 g/l CaCl_2_ × 2 H_2_O, 0.11 g/l K_2_HPO_4_, 0.2 g/l KH_2_PO_4_, 18.0 g/l NaCl, 0.3 g/l yeast extract, 2.5 g/l NaHCO_3,_ a 100 × trace element solution (1.5 g/l nitrilotriacetic acid, 3 g/l MgSO_4_ × 7 H_2_O, 0.45 g/l MnSO_4_ × 2 H_2_O, 1 g/l NaCl, 0.1 g/l FeSO_4_ × 7 H_2_O, 0.18 g/l CoSO_4_ × 6 H_2_O, 0.1 g/l CaCl_2_ × 2 H_2_O, 0.18 g/l ZnSO_4_ × 7 H_2_O, 0.01 g/l CuSO_4_ × 5 H_2_O, 0.02 g/l KAI(SO_4_)_2_ × 12 H_2_O, 0.01 g/l H_3_BO_3_, 0.01 g/l Na_2_WO_4_ × 2 H_2_O, 0.01 g/l Na_2_MoO_4_ × 2 H_2_O, 0.025 g/l NiCl_2_ × 6 H_2_O, 0.01 g/l Na_2_SeO_3_) and 0.5 ml/l 0.2% resazurin. Before inoculating, sterile anoxic solutions of cysteine (0.5 mM), Na_2_S (0.5 mM), MgSO_4_ (16 mM), L‐lactate (35 mM) or 2‐methoxyphenol (2‐MP; 7.5 to 30 mM) were added to the autoclaved medium. Cultivation was performed at 80°C. Next to 2‐methoxyphenol also 7.5 mM 2‐methoxybenzoate, 3,4,5‐trimethoxybenzoate, 2,6‐dimethoxyphenol, 3,5‐dimethoxy‐4‐hydroxycinnamic acid, 4‐methoxyphenylacetic acid, methoxyhydroquinone, methyl 2‐methoxybenzoate and 15 mM methanol were tested as growth substrate. Growth could only be observed with certainty for 2‐methoxyphenol, 2,6‐dimethoxyphenol, methoxyhydroquinone and 2‐methoxybenzoate (Supporting Information Fig. S2). Furthermore, it was tested if *A. fulgidus* can grow without CO_2_ with lactate or 2‐MP as substrate. Medium without CO_2_ was prepared as described above but leaving out NaHCO_3_, adding 4.6 g/l piperazine‐N,N′‐bis(2‐ethanesulfonic acid) (PIPES), adjusting the pH to 6.9 and gassing with N_2_ instead of N_2_‐CO_2_.

### Following growth and substrate consumption of *A. fulgidus*

Growth was followed by measuring the optical density at 600 nm (OD_600_) with a Cary 60 UV–Vis spectrophotometer (Agilent Technologies, USA) at distinct time points. To prevent interference of the absorbance of resazurin, which is present in the medium, sodium dithionite was added to the cuvettes before measuring OD_600_. For all growth conditions, triplicates were used and those triplicates were also used for analysis of substrate consumption/product formation which is described in the following section.

#### Determination of CO_2_ and H_2_

The CO_2_ and H_2_ concentration in the headspace was measured by gas chromatography with a gas chromatograph (Hewlett Packard 5890a, Agilent Technologies, Santa Clara, CA, USA) equipped with a Porapak Q 100/120 mesh and a thermal conductivity detector (TCD) using N_2_ as carrier gas. Each measurement was performed by injection of 50 μl headspace gas with a gas‐tight syringe. CO_2_ concentrations were corrected for the volume removed from the bottle due to OD_600_ measurements. No H_2_ production or consumption could be observed.

#### Determination of methane

To analyse if the cultures produce methane, 50 μl headspace volume were injected with a gas‐tight glass syringe (Hamilton, Reno, NE) into an Agilent 6890 series gas chromatograph coupled to a Agilent 5975C mass spectrometer with triple‐Axis detector (GC–MS) (Agilent, Santa Clara, CA) equipped with a Porapak Q column heated at 80°C. No methane production could be observed.

#### Determination of 2‐methoxyphenol and 2‐hydroxyphenol

To measure concentrations of 2‐methoxyphenol and 2‐hydroxyphenol by HPLC an Agilent 1100 HPLC system equipped with a diode array detector (detecting wavelength 260 nm) and a Merck C‐18e column (250 mm × 4.6 mm, 5 μm particle size) was used. The flow rate was 0.75 ml/min and a linear gradient was applied: 75% trifluoroacetic acid (TFA) (0.1% in water), 25% acetonitrile to 50% TFA (0.1% in water), 50% acetonitrile in 15 min. A solution of 2‐methoxyphenol and 2‐hydroxyphenol in water (0.1 mg/ml) was used as standard. Twenty microlitres of sample was used for injection.

#### Acetate determination

For the determination of acetate concentrations, a slightly modified method of Kage *et al*. (2004) was used. Two hundred microlitres of pentafluorobenzylbromide (PFBBr) solution (100 mM in acetone) and 40 μl phosphate buffer (0.5 M, pH 6.8) were added to 40 μl sample, mixed and incubated for 1 h at 60°C in an eppendorf tube with safety cap. After cooling down to room temperature, 400 μl hexane (containing 50 μg/ml methylstearate as internal standard) was added and vortexed for 1 min. Thereafter, the sample was centrifuged at 15 000 × *g* for 1 min. From the hexane layer (top), 100 μl was transferred to a GC vial. One microlitre of the sample was used for analysis by GCMS. To determine precise acetate concentrations, a calibration curve was made. A stock solution of 100 mM sodium acetate in water was prepared and diluted in a range between 1 and 40 mM acetate. Of each of these dilutions, 40 μl was used for preparation of a calibration curve. Samples were derivatized as described above for the samples. For analysis of PFBBr derivatives, an Agilent 7890A gas chromatograph equipped with an autosampler (7693A) and with a JMS‐T100GCv JEOL (JEOL) mass spectrometer was used. The gas chromatograph contains a HP‐5MS column 30 m × 0.25 mm × 0.25 μm. The following conditions were applied for GC‐TOF‐MS acetate analysis: The oven temperature was set to 50°C for 2 min, followed by a temperature gradient of 20°C/min to 300°C for 1 min. The split ratio was 1:10 and the detector voltage 2000 V. Peaks were detected by total ion current (TIC) and a selected trace with mass range m/z 239.9–240.1 used for detection and quantification of the acetate‐PFB derivative. Data were evaluated with Xcalibur (version 2.1, Thermo Scientific) after conversion/export to net CDF by MassCenter JEOL.

#### Sulfide determination

Ten microlitres of samples were used immediately for sulfide determination with the methylene blue method by using a sulfide reagent set (HACH, USA; method 8131) according to the manufacturer's instructions. Samples were diluted 100‐fold. For calculation of the sulfide concentration the Henrys law constant of 0.001 (mol s^2^ m^−2^ kg^−1^) was used (Sander, 2015). A factor of 2.9 was calculated with the help of this constant and by adjusting for the 80°C incubation temperature to calculate the sulfide concentration in the headspace with help of the following equation *KRT* = *c*(liquid)/*c*(gasphase), where *K* is Henrys law constant, *R* the gas constant and *K* the temperature.

#### Sulfate determination

Twenty microlitres of samples were used to measure sulfate concentrations with a Sulfate Assay kit (MAK132, Sigma‐Aldrich) according to the manufacturer's instructions. Samples were diluted 10‐fold.

#### Formate determination

Five microlitres of samples were used for measuring formate with a Formate Assay kit (MAK059, Sigma‐Aldrich) according to the manufacturer's instructions. Samples were diluted 10‐fold.

#### Lactate determination

Lactate was measured after the method described by Borshchevskaya *et al*. (2016). Twenty‐five microlitres of sample was mixed with 1 ml 0.2% iron (III) chloride hexahydrate. Using 10 g/l acetate and formate instead of sample leads to Abs_390_ nm value similar to those of the medium background, showing that those compounds do not interfere with the assay. With help of this assay, it was observed that about 23 mM (1.2 mmol per bottle) lactate out of 42 mM (2.1 mmol per bottle) lactate were consumed during the growth of *A. fulgidus*.

### Resting cell experiment

*A. fulgidus* cells grown in 50 ml medium (see above) with 12 mM 2‐MP or 35 mM lactate as substrate were harvested anaerobically in the exponential phase and washed with stabilization buffer (2 mM KH_2_PO_4_/K_2_HPO_4_, 2 mM MgSO_4_, 400 mM NaCl, 200 mM sucrose, pH 6.8, gassed with N_2_). The cell pellets were resuspended in 40 ml stabilization buffer (see above) and transferred into 120 ml anaerobic glass bottles. The cultures were incubated for 30 min at 80°C. Afterwards, 6 mM 2‐MP or 17 mM lactate and 8 mM MgSO_4_ were added and the cultures were incubated for 5 h at 80°C. The CO_2_ gas produced by the cultures was analysed every hour by injecting 50 μl headspace volume with a gas‐tight glass syringe (Hamilton, Reno, NE) into an Agilent 6890 series gas chromatograph coupled to a mass spectrometer (GC–MS) (Agilent, Santa Clara, CA) equipped with a Porapak Q column heated at 80°C. For calculating the CO_2_ in the culture headspace, a calibration curve was generated by injecting different volumes of calibration gas (Linde Gas Benelux) that contained 1% CO_2_ and 1% CH_4_ into the GC–MS. The CO_2_ values (in %) were corrected for the CO_2_ in the medium in form of HCO_3_^−^ by measuring the headspace CO_2_ in 40 ml buffer before and after acidification with HCl. The experiment was performed in triplicate. Values for CO_2_ production were adjusted to the protein content of each sample. For the protein determination, 500 μl of cells were sampled at *t*_0_ and boiled for 15 min at 110°C. Afterwards, 2 ml −20°C cold acetone was added and the samples incubated at −20°C for 1 h. The samples were centrifuged at 20 000 × *g* for 15 min, the supernatant removed and the pellets dried for 30 min at room temperature. After resuspending the pellets in 50 μl H_2_O, the protein content was measured by the Bradford Protein Assay (5000006, Bio‐Rad) according to the manufacturer's instructions.

### Metabolite analysis of *A. fulgidus* cells grown under different conditions

*A. fulgidus* was grown in triplicates in 50 ml medium (see above) with either 12 mM 2‐MP (Mp), 35 mM lactate (Lac) or 12 mM 2‐MP plus 35 mM lactate (M + L). As a control, a culture without substrate was used (no change in OD_600_). Cells were harvested aerobically in the exponential phase (OD_600_ MP: 0.06, Lac: 0.13, M + L: 0.15) at 20 000 × *g* and 4°C for 15 min. Pellets were washed with 1 ml cold PBS and centrifuged for 1 min at 12 000 × *g*. The remaining pellet was lysed using 500 μl of H_2_O:methanol:acetonitrile(40:40:20, V:V:V). After centrifugation for 1 min at 12 000 × g, the supernatant was transferred into a fresh tube and stored at −20°C until metabolite analysis. Aqueous normal phase metabolomics was performed on 2 μl sample using a 1290 Infinity II LC system coupled to a 6546 Q‐ToF MS (Agilent Technologies) as previously described (Jansen *et al*., 2020). In brief, samples were injected onto a Diamond Hydride Type C column (Cogent) and separated using a gradient of water and acetonitrile with 0.2% formic acid. Detection was performed in the negative ionization mode from m/z 50–1200. For the detection of glutamate, samples were diluted 20‐fold in acetonitrile:methanol:water (40:40:20, V:V:V) to overcome sodium‐related interferences. Metabolites were identified based on MS fragmentation and quantified using Qualitative Analysis 10.0 (Agilent Technologies). Metabolite signals were normalized for OD_600_.

### RNA isolation from *A. fulgidus* cells

Cultures for RNA extractions/transcriptomics were grown in 250 ml medium with either 10 mM 2‐MP or 35 mM lactate. Cells were harvested anaerobically in the exponential phase at 10 000 × *g*, 20 min and 4°C. The pellet was frozen in liquid nitrogen and stored at −80°C until RNA isolation. RNA isolation was performed with the RiboPure‐Bacteria Kit (Thermo Fischer Scientific) according to the manufacturer's instructions. Quantity and quality of RNA from lactate and 2‐MP grown cells (in triplicates) were checked with an Agilent 2100 Bioanalyzer and the RNA Integrity Number was between 7.2 and 8.2.

### Transcriptome sequencing and analyses

For library preparation, the TruSeq® Stranded mRNA Library Prep protocol (Illumina, San Diego, CA, USA) was used according to the manufacturer's instructions. Total RNA was used for library preparation. The library concentration measured with a Qubit fluorometer and the average fragment size obtained with the Agilent 2100 Bioanalyzer were used to calculate the correct dilution factor required for normalization of the library. After dilution to 4 nM and denaturation using the Denature and Dilute Libraries Guide (Illumina, San Diego, CA), the library was sequenced using an Illumina MiSeq machine (San Diego, CA) to generate 150 bp single‐end reads.

To analyse transcriptomic data, raw reads from the MiSeq platform were initially trimmed based on a quality limit of 0.02 and minimal length of 50 bp using the CLC Genomics Workbench 12 (Qiagen, Aarhus, Denmark). Next, RNA reads were mapped to the draft genome of *A. fulgidus* DSM 4304 AE000782.1 (insertion cost, 3; deletion cost, 3; length fraction, 0.8; similarity fraction, 0.95). Further analysis was conducted with RStudio using R package ‘Deseq2’ (Love *et al*., 2014). The gene expression values were expressed as RPKM (Reads per kilo base of exon model per million mapped reads) and log_2_fold change values. For the generation of the heat map, RPKM values were imported into RStudio v. 1.2.5033 (using R v. 4.0.3), and the packages RColorBrewer v. 1.1.2, gplots v. 3.1.1 and vegan v. 2.5.6 were used, with the function ‘heatmap.2’.

### Protein sequence analyses

We identified relevant archaeal sequences via BlastP using *A. fulgidus* amino acid sequences of MtoABCD, blasting them against the non‐redundant blast database and we selected sequences with at least 35% amino acid identity coming from one same genome (*Candidatus* Bathyarchaeota archaeon SpSt‐709: DTEF01, *Candidatus* Verstraetearchaeota archaeon SJ3.Bin21: JAAOZJ01, *Candidatus* Nezhaarchaeota archaeon WYZ‐LMO8: QNVF01, *Candidatus* Lokiarchaeota archaeon Hel_238_bin90: JABXKC01, *Candidatus* Helarchaeota archaeon CR_Bin_143: JABXJV01, *Candidatus* Korarchaeota archaeon B41_G2: QMVX01). Improved protein annotation was obtained via NCBI blast analysis (https://blast.ncbi.nlm.nih.gov/Blast.cgi?PAGE=Proteins). The phylogenetic tree was generated via multiple alignment of amino acid sequences retrieved from NCBI with MUSCLE (Edgar, 2004) v. 3.8.31 (sequence accession numbers are provided in the tree). Alignment columns were stripped with trimAI (Capella‐Gutiérrez *et al*., 2009) v. 1.4.rev22 using the ‐gappyout option, and the tree was built with FastTree (Price *et al*., 2010) v.2.1.10 using the Jones‐Taylor‐Thorton maximum likelihood model. The tree was visualized with iToL (Letunic and Bork, 2019) v.4 and edited on Adobe Illustrator CC 2018 (San Jose, California, USA).

## Supporting information

**Appendix S1**: Supporting information.

## Data Availability

The transcriptomics data have been deposited at the Sequence Read Archive (SRA) from NCBI with BioProject accession number PRJNA695423 under the following link: https://www.ncbi.nlm.nih.gov/sra/PRJNA695423.
