# Supplementary material for: A novel methoxydotrophic metabolism discovered in the hyperthermophilic archaeon Archaeoglobus fulgidus
Source: Environ Microbiol. 2021 May 5;23(7):4017–33. doi: 10.1111/1462-2920.15546 (PMC8359953; doi:10.1111/1462-2920.15546)
Supplement: Supplementary file 1 — Appendix S1: Supporting information. [file EMI-23-4017-s001.docx]

**Supplementary Material for**

A novel methoxydotrophic metabolism discovered in the hyperthermophilic archaeon *Archaeoglobus fulgidus*

Cornelia U. Welte^abc^, Rob de Graaf^a^, Paula Dalcin Martins^a^, Robert S. Jansen^a^, Mike S.M. Jetten^abc^, Julia M. Kurth*^ac^

^a^ Department of Microbiology, Institute for Water and Wetland Research, Radboud University, Heyendaalseweg 135, 6525 AJ Nijmegen, The Netherlands

^b^ Netherlands Earth System Science Center, Utrecht University, Heidelberglaan 2, 3584 CS Utrecht, The Netherlands

^c^ Soehngen Institute of Anaerobic Microbiology, Radboud University, Heyendaalseweg 135, 6525 AJ Nijmegen, The Netherlands

*Corresponding author: Department of Microbiology, Institute for Water and Wetland Research, Radboud University, Heyendaalseweg 135, 6525 AJ Nijmegen, The Netherlands. E-mail: j.kurth@science.ru.nl

**
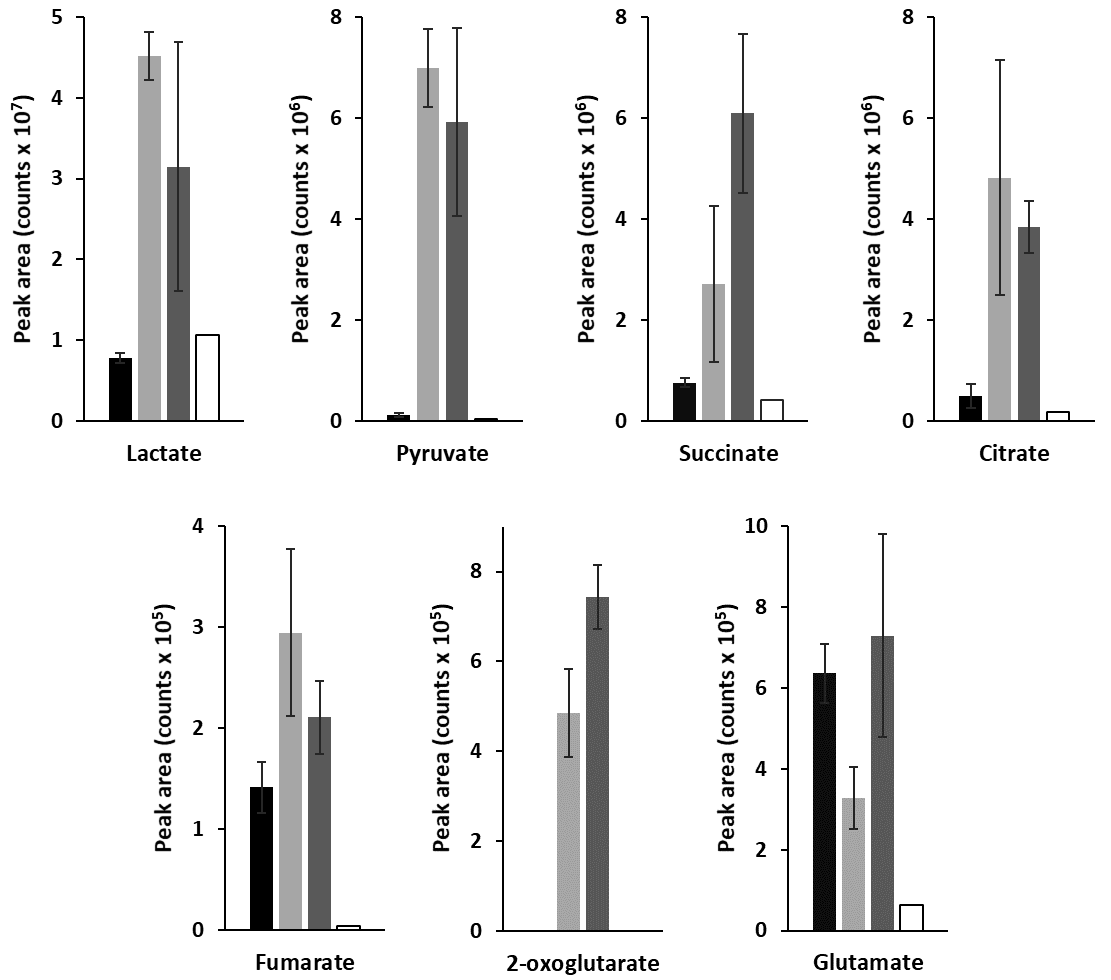
**

**SI figure 1: Metabolite analysis of *A. fulgidus* cells grown under different conditions.** *A. fulgidus* was grown on 12 mM 2-methoxyphenol (black), 35 mM lactate (light grey), 12 mM 2-methoxyphenol plus 35 mM lactate (dark grey) and without substrate (white). Samples were analysed by Q-ToF MS.

**
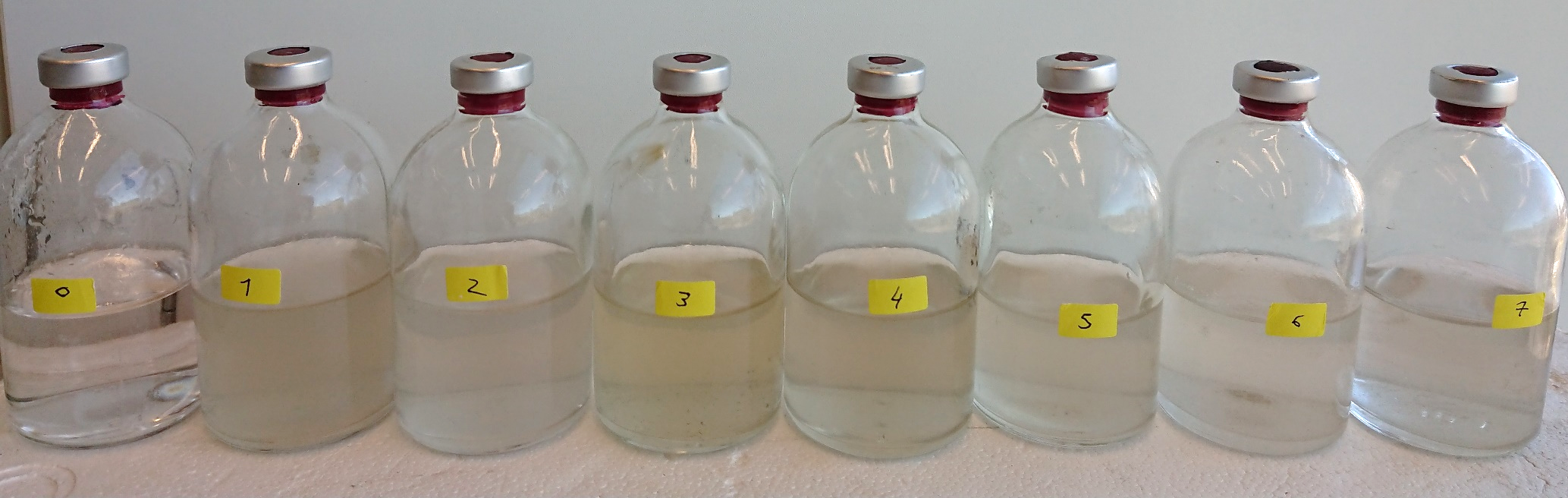
**

**SI figure 2: Growth of *A. fulgidus* on different substrates.** *A. fulgidus* was incubated in medium containing different substrates plus sulfate for about 3 days at 80°C. Growth was detected on 2-methoxyphenol, 2,6-dimethoxyphenol, methoxyhydroquinone and 2-methoxybenzoate by OD_600_ measurements and microscopy. For substrates such as 3,5-dimethoxy-4-hydroxycinnamic acid and 3,4,5-trimethoxybenzoate a slight increase in OD_600_ was observed but no growth could be detected by using microscopy. 0: *A. fulgidus* medium, 1: lactate (OD_600_: 0.237), 2: 2-methoxyphenol (OD_600_: 0.12), 3: 2,6-dimethoxyphenol (OD_600_: 0.141), 4: methoxyhydroquinone (OD_600_: 0.101), 5: 2-methoxybenzoate (OD_600_: 0.136, 6: 3,5-dimethoxy-4-hydroxycinnamic (OD_600_: 0.055), 7: 3,4,5-trimethoxybenzoate (OD_600_: 0.050).

**SI table 1: RPKM and log_2_ fold change values with corresponding *p*_adj_ values of selected genes corresponding to processes in Figure 7.** Log_2_ fold change values are shown for *A. fulgidus* grown on 2-methoxyphenol (MP) versus lactate (Lac). RPKM: Reads Per Kilobase Million, *p*_adj_: adjusted p value (calculated with DESeq2 (Love *et al.*, 2014)). The genes with adjusted p-value of NA have less mean normalized counts than the optimal threshold. Additionally, genes involved in lipid metabolism are shown.

| **Locus tag** | **Annotation** | **RPKM Lac** | **RPKM MP** | **log_2_ fold change** | ***p*_adj_** |
| --- | --- | --- | --- | --- | --- |
|  | **Methyl transfer** |  |  |  |  |
| AF_0006 | cobalamin-binding protein, MtoC | 221.7 | 13460.7 | 5.7 | 0.000 |
| AF_0007 | O-demethylase, MtoB | 68.6 | 4719.1 | 5.9 | 0.000 |
| AF_0008 | MFS transporter | 8.8 | 314.4 | 5.1 | 0.000 |
| AF_0009 | MtrH-like methyl transferase, MtoA | 43.6 | 1061.4 | 4.5 | 0.000 |
| AF_0010 | Corrinoid activation protein, MtoD | 41.1 | 861.8 | 4.0 | 0.000 |
| AF_0011 | cobalamin-binding protein, MtoC2 | 17.6 | 308.8 | 4.0 | 0.000 |
| AF_0012 | O-demethylase, MtoB2 | 8.6 | 113.3 | 3.6 | 0.000 |
| AF_0013 | MFS transporter | 1.7 | 28.5 | 4.0 | 0.000 |
|  | **Acetyl-CoA pathway** |  |  |  |  |
| AF_0177 | Formylmethanofuran dehydrogenase subunit FwdE | 38.0 | 154.6 | 2.0 | 0.000 |
| AF_1644 | Formylmethanofuran dehydrogenase subunit FwdF | 324.3 | 236.1 | -0.6 | 0.020 |
| AF_1649 | Formylmethanofuran dehydrogenase subunit FwdG | 82.2 | 116.3 | 0.4 | 0.399 |
| AF_1650 | Formylmethanofuran dehydrogenase subunit FwdB-1 | 103.4 | 134.0 | 0.2 | 0.655 |
| AF_1651 | Formylmethanofuran dehydrogenase subunit FwdD-1 | 21.8 | 38.3 | 0.7 | 0.207 |
| AF_1928 | Formylmethanofuran dehydrogenase subunit FwdD-2 | 544.6 | 509.6 | -0.4 | 0.362 |
| AF_1929 | Formylmethanofuran dehydrogenase subunit FwdB-2 | 454.7 | 464.8 | -0.2 | 0.587 |
| AF_1930 | Formylmethanofuran dehydrogenase subunit FwdA | 420.4 | 430.5 | -0.2 | 0.630 |
| AF_1931 | Formylmethanofuran dehydrogenase subunit FwdC | 1104.3 | 1196.2 | -0.1 | 0.804 |
| AF_2073 | formylmethanofuran-tetrahydromethanopterin formyl-transferase Ftr-1 | 105.3 | 107.3 | -0.1 | 0.727 |
| AF_2207 | formylmethanofuran-tetrahydromethanopterin formyl-transferase Ftr-2 | 264.2 | 223.1 | -0.4 | 0.158 |
| AF_1935 | methenyltetrahydromethanopterin cyclohydrolase Mch | 295.3 | 268.0 | -0.3 | 0.346 |
| AF_0714 | methylenetetrahydromethanopterin dehydrogenase Mtd | 416.0 | 380.2 | -0.4 | 0.236 |
| AF_1066 | 5,10-methylenetetrahydromethanopterin reductase Mer-1 | 402.0 | 287.1 | -0.7 | 0.017 |
| AF_1196 | 5,10-methylenetetrahydromethanopterin reductase Mer-2 | 11.0 | 29.6 | 1.3 | 0.000 |
| AF_0376 | acetyl-CoA decarbonylase/synthase CODH/ACS complex subunit CdhE | 251.2 | 321.1 | 0.2 | 0.511 |
| AF_0377 | acetyl-CoA decarbonylase/synthase CODH/ACS complex subunit CdhD | 288.8 | 355.6 | 0.2 | 0.578 |
| AF_0379 | acetyl-CoA decarbonylase/synthase CODH/ACS complex subunit CdhC | 494.9 | 608.8 | 0.1 | 0.643 |
| AF_1100 | acetyl-CoA decarbonylase/synthase CODH/ACS complex subunit CdhA-1 | 326.8 | 157.6 | -1.3 | 0.000 |
| AF_1101 | acetyl-CoA decarbonylase/synthase CODH/ACS complex subunit CdhB-1 | 131.4 | 78.7 | -0.9 | 0.006 |
| AF_2397 | acetyl-CoA decarbonylase/synthase CODH/ACS complex subunit CdhA-2 | 778.6 | 759.9 | -0.3 | 0.475 |
| AF_2398 | acetyl-CoA decarbonylase/synthase CODH/ACS complex subunit CdhB-2 | 453.7 | 491.4 | -0.1 | 0.886 |
|  | **Acetate metabolism** |  |  |  |  |
| AF_0197 | acetyl-CoA synthetase Acs-1 | 2.8 | 3.2 | 0.2 | 0.745 |
| AF_0366 | acetyl-CoA synthetase Acs-2 | 4.1 | 28.0 | 2.6 | 0.000 |
| AF_0677 | acetyl-CoA synthetase Acs-3 | 8.1 | 14.7 | 0.7 | 0.064 |
| AF_0975 | acetyl-CoA synthetase Acs-4 | 18.3 | 147.6 | 2.9 | 0.000 |
| AF_0976 | acetyl-CoA synthetase Acs-5 | 15.1 | 130.8 | 3.0 | 0.000 |
| AF_1211 | acetate-CoA ligase Acl | 16.8 | 9.9 | -0.9 | 0.018 |
| AF_1938 | acetate-CoA ligase Acl | 97.3 | 52.2 | -1.1 | 0.000 |
|  | **Pyruvate metabolism** |  |  |  |  |
| AF_1699 | pyruvate ferredoxin oxidoreductase subunit PorG | 1097.3 | 364.8 | -1.8 | 0.000 |
| AF_1700 | pyruvate ferredoxin oxidoreductase subunit PorD | 178.7 | 61.0 | -1.7 | 0.000 |
| AF_1701 | pyruvate ferredoxin oxidoreductase subunit PorA | 431.0 | 176.8 | -1.4 | 0.000 |
| AF_1702 | pyruvate ferredoxin oxidoreductase subunit PorB | 686.7 | 290.5 | -1.4 | 0.000 |
| AF_1449 | pyruvate formate lyase subunit PflD | 21.3 | 20.9 | -0.1 | 0.810 |
| AF_1450 | pyruvate formate lyase subunit PflC | 6.8 | 9.7 | 0.5 | 0.407 |
| AF_1961 | pyruvate formate lyase subunit PflX | 81.6 | 98.2 | 0.1 | 0.728 |
| AF_0117 | pyruvate formate lyase activating enzyme Act-1 | 31.6 | 31.2 | -0.1 | 0.797 |
| AF_0918 | pyruvate formate lyase activating enzyme Act-2 | 72.5 | 45.1 | -0.7 | 0.016 |
| AF_1330 | pyruvate formate lyase activating enzyme Act-3 | 38.0 | 96.1 | 1.2 | 0.000 |
| AF_2278 | pyruvate formate lyase activating enzyme Act-4 | 26.6 | 36.6 | 0.3 | 0.519 |
|  | **Lactate metabolism** |  |  |  |  |
| AF_0394 | D-lactate dehydrogenase Dld | 17.3 | 34.3 | 0.8 | 0.030 |
| AF_0808 | D-lactate dehydrogenase Dld | 166.2 | 261.3 | 0.8 | NA |
| AF_0807 | L-lactate dehydrogenase LldD | 168.6 | 126.7 | -0.3 | 0.867 |
| AF_0809 | lactate utilization protein; putative L-lactate dehydrogenase subunit LldF | 74.9 | 158.2 | 1.3 | 0.487 |
| AF_0810 | lactate utilization protein, putative L-lactate dehydrogenase subunit LldG | 133.0 | 170.8 | 0.5 | NA |
| AF_0811 | L-lactate dehydrogenase LldG | 150.5 | 204.8 | 0.6 | NA |
| AF_0806 | L-lactate permease LctP | 34.4 | 331.6 | 3.4 | NA |
|  | **TCA cycle** |  |  |  |  |
| AF_0220 | pyruvate carboxylase PycA | 31.2 | 169.6 | 2.5 | 0.000 |
| AF_1486 | phosphoenolpyruvate carboxylase | 3.9 | 7.1 | 0.8 | 0.122 |
| AF_1252 | oxaloacetate decarboxylase subunit OadA | 138.9 | 158.0 | 0.0 | 0.989 |
| AF_0855 | malate dehydrogenase MdhA | 101.5 | 142.6 | 0.3 | 0.386 |
| AF_1727 | malate oxidoreductase Mae | 50.0 | 24.7 | -1.3 | 0.000 |
| AF_0628 | 3-isopropylmalate dehydrogenase LeuB | 106.9 | 102.6 | -0.2 | 0.557 |
| AF_0629 | 3-isopropylmalate dehydratase LeuD | 199.0 | 195.1 | -0.2 | 0.567 |
| AF_1098 | fumarate hydratase Fum-1 | 8.2 | 33.3 | 1.8 | 0.000 |
| AF_1099 | fumarate hydratase Fum-2 | 11.3 | 42.8 | 1.7 | 0.000 |
| AF_0681 | succinate dehydrogenase subunit SdhA | 74.7 | 195.0 | 1.2 | 0.000 |
| AF_0682 | succinate dehydrogenase subunit SdhB | 10.6 | 37.9 | 1.7 | 0.000 |
| AF_0683 | succinate dehydrogenase subunit SdhC | 13.0 | 29.3 | 1.0 | 0.049 |
| AF_0684 | succinate dehydrogenase subunit SdhD | 17.9 | 59.0 | 1.6 | 0.000 |
| AF_1539 | succinyl-CoA synthetase SucD-1 | 7.1 | 12.6 | 0.6 | 0.207 |
| AF_1540 | succinyl-CoA synthetase SucC-1 | 12.8 | 15.5 | 0.0 | 0.936 |
| AF_2185 | succinyl-CoA synthetase SucD-2 | 39.6 | 56.8 | 0.4 | 0.335 |
| AF_2186 | succinyl-CoA synthetase SucC-2 | 42.7 | 62.6 | 0.3 | 0.335 |
| AF_0468 | 2-ketoglutarate ferredoxin oxidoreductase subunit KorB | 42.2 | 835.4 | 4.2 | 0.000 |
| AF_0469 | 2-ketoglutarate ferredoxin oxidoreductase subunit KorA | 35.3 | 505.3 | 3.8 | 0.000 |
| AF_0470 | 2-ketoglutarate ferredoxin oxidoreductase subunit KorD | 53.5 | 552.8 | 3.3 | 0.000 |
| AF_0471 | 2-ketoglutarate ferredoxin oxidoreductase subunit KorG | 17.0 | 168.0 | 3.2 | 0.000 |
| AF_0749 | 2-oxoglutarate/2-oxoacid ferredoxin oxidoreductase subunit OrA | 72.3 | 54.3 | -0.5 | 0.052 |
| AF_0750 | 2-oxoglutarate/2-oxoacid ferredoxin oxidoreductase subunit OrB | 22.2 | 19.2 | -0.3 | 0.465 |
| AF_0952 | glutamate synthase GltB-1 | 375.9 | 180.7 | -1.2 | 0.000 |
| AF_0953 | glutamate synthase GltB-2 | 273.7 | 114.1 | -1.4 | 0.000 |
| AF_0954 | glutamate synthase GltB-3 | 356.0 | 145.2 | -1.5 | 0.000 |
| AF_2295 | aspartate aminotransferase family protein, decarboxylase | 4.5 | 54.3 | 3.5 | 0.000 |
| AF_2366 | aspartate aminotransferase AspB-1 | 59.2 | 40.9 | -0.7 | 0.022 |
| AF_2129 | aspartate aminotransferase AspB-2 | 192.4 | 155.1 | -0.5 | 0.106 |
| AF_1623 | aspartate aminotransferase AspB-3 | 129.8 | 83.6 | -0.7 | 0.006 |
| AF_0409 | aspartate aminotransferase AspB-4 | 49.0 | 43.6 | -0.4 | 0.270 |
| AF_1417 | aspartate aminotransferase AspC | 262.1 | 150.0 | -1.0 | 0.000 |
| AF_0647 | isocitrate dehydrogenase Icd | 140.0 | 125.5 | -0.3 | 0.253 |
| AF_1963 | aconitase Acn | 208.7 | 107.4 | -1.1 | 0.000 |
| AF_1340 | citrate synthase citZ | 143.5 | 60.2 | -1.4 | 0.000 |
|  | **Sulfate metabolism** |  |  |  |  |
| AF_1667 | sulfate adenylyltransferase Sat | 1578.5 | 1332.8 | -0.4 | 0.198 |
| AF_1669 | adenylylsulfate reductase subunit AprB | 1408.9 | 846.4 | -0.8 | 0.006 |
| AF_1670 | adenylylsulfate reductase subunit AprA | 2546.3 | 1311.6 | -1.1 | 0.000 |
| AF_0423 | dissimilatory sulfite reductase subunit DsrA | 1632.2 | 1687.7 | -0.2 | 0.664 |
| AF_0424 | dissimilatory sulfite reductase subunit DsrB | 1673.5 | 1923.8 | 0.0 | 0.998 |
| AF_0425 | dissimilatory sulfite reductase subunit DsrD | 535.7 | 722.2 | 0.2 | 0.506 |
| AF_2228 | dissimilatory sulfite reductase related protein DsrC | 727.8 | 629.7 | -0.4 | 0.254 |
| AF_0661 | quinone-modifying oxidoreductase subunit QmoC | 128.0 | 198.4 | 0.5 | 0.113 |
| AF_0662 | quinone-modifying oxidoreductase subunit QmoB | 497.8 | 601.9 | 0.1 | 0.775 |
| AF_0663 | quinone-modifying oxidoreductase subunit QmoA | 262.6 | 317.4 | 0.1 | 0.856 |
| AF_0499 | molybdopterin oxidoreductase subunit DsrO | 164.9 | 304.7 | 0.7 | 0.010 |
| AF_0500 | molybdopterin oxidoreductase subunit DsrP | 66.0 | 148.1 | 1.1 | 0.001 |
| AF_0501 | molybdopterin oxidoreductase subunit DsrM | 164.6 | 244.2 | 0.4 | 0.159 |
| AF_0502 | molybdopterin oxidoreductase subunit DsrK | 134.8 | 221.9 | 0.6 | 0.032 |
| AF_0503 | molybdopterin oxidoreductase subunit DsrJ | 64.8 | 111.9 | 0.7 | 0.053 |
| AF_0543 | Putative membrane complex subunit DsrK | 469.2 | 616.2 | 0.3 | 0.417 |
| AF_0544 | Putative membrane complex subunit DsrK | 386.9 | 504.4 | 0.3 | 0.448 |
| AF_0545 | Putative membrane complex subunit DsrM | 670.9 | 793.7 | 0.1 | 0.842 |
| AF_0546 | Putative membrane complex subunit DsrM | 8.6 | 8.5 | -0.3 | 0.683 |
| AF_0547 | Putative membrane complex subunit DsrK | 21.3 | 19.1 | -0.3 | 0.562 |
| AF_0755 | Putative heterodisulfide reductase subunits E and D | 16.0 | 14.4 | -0.3 | 0.470 |
|  | **Energy metabolism & regeneration of reducing equivalents** |  |  |  |  |
| AF_1823 | F_420_H_2_:quinone oxidoreductase subunit FqoJ | 74.8 | 50.4 | -0.8 | 0.020 |
| AF_1824 | F_420_H_2_:quinone oxidoreductase subunit FqoK | 84.5 | 62.9 | -0.5 | 0.170 |
| AF_1825 | F_420_H_2_:quinone oxidoreductase subunit FqoM | 109.8 | 108.0 | -0.1 | 0.749 |
| AF_1826 | F_420_H_2_:quinone oxidoreductase subunit FqoL | 135.9 | 109.5 | -0.4 | 0.213 |
| AF_1827 | F_420_H_2_:quinone oxidoreductase subunit FqoN | 103.3 | 100.2 | -0.1 | 0.821 |
| AF_1828 | F_420_H_2_:quinone oxidoreductase subunit FqoA | 103.9 | 103.2 | -0.1 | 0.873 |
| AF_1829 | F_420_H_2_:quinone oxidoreductase subunit FqoB/C | 266.7 | 188.4 | -0.6 | 0.047 |
| AF_1830 | F_420_H_2_:quinone oxidoreductase subunit FqoD | 216.5 | 157.8 | -0.5 | 0.054 |
| AF_1831 | F_420_H_2_:quinone oxidoreductase subunit FqoH | 62.2 | 65.7 | 0.0 | 0.968 |
| AF_1832 | F_420_H_2_:quinone oxidoreductase subunit FqoI | 169.4 | 129.5 | -0.5 | 0.110 |
| AF_1833 | F_420_H_2_:quinone oxidoreductase subunit FqoF | 120.6 | 116.0 | -0.2 | 0.617 |
| AF_1375 | soluble heterodisulfide reductase subunit HdrB | 5.9 | 47.1 | 2.8 | 0.000 |
| AF_1376 | soluble heterodisulfide reductase subunit HdrC | 1.4 | 12.6 | 3.0 | 0.000 |
| AF_1377 | soluble heterodisulfide reductase subunit HdrA | 1.8 | 49.3 | 4.5 | 0.000 |
| AF_1371 | F_420_-nonreducing hydrogenase subunit VhtD-1 | 4.1 | 14.9 | 1.8 | 0.010 |
| AF_1372 | F_420_-nonreducing hydrogenase subunit VhuA | 4.6 | 30.9 | 2.7 | 0.000 |
| AF_1373 | F_420_-nonreducing hydrogenase subunit VhuG | 3.8 | 36.8 | 3.2 | 0.000 |
| AF_1374 | F_420_-nonreducing hydrogenase subunit VhuD | 2.3 | 35.1 | 3.8 | 0.000 |
| AF_1378 | F_420_-nonreducing hydrogenase subunit VhtD-2 | 1.3 | 58.9 | 5.5 | 0.000 |
| AF_1379 | quinone-reactive Ni/Fe-hydrogenase B-type cytochrome subunit HydC; Putative F_420_-nonreducing hydrogenase subunit VhtC | 7.0 | 170.5 | 4.4 | 0.000 |
| AF_1380 | F_420_-nonreducing hydrogenase VhtA | 2.2 | 126.6 | 5.7 | 0.000 |
| AF_1381 | F_420_-nonreducing hydrogenase VhtG | 1.4 | 139.2 | 6.5 | 0.000 |
| AF_1158 | V/A-type H^+^/Na^+^-transporting ATPase subunit AtpG/H | 1173.5 | 1530.7 | 0.2 | 0.642 |
| AF_1159 | V/A-type H^+^/Na^+^-transporting ATPase subunit AtpI | 263.1 | 391.0 | 0.5 | 0.086 |
| AF_1160 | V/A-type H^+^/Na^+^-transporting ATPase subunit AtpK | 243.4 | 140.3 | NA | NA |
| AF_1162 | V/A-type H^+^/Na^+^-transporting ATPase subunit AtpK-2 | 248.7 | 142.5 | NA | NA |
| AF_1163 | V/A-type H^+^/Na^+^-transporting ATPase subunit AtpE | 654.5 | 641.8 | -0.1 | 0.781 |
| AF_1164 | V/A-type H^+^/Na^+^-transporting ATPase subunit AtpC | 257.3 | 282.0 | 0.1 | 0.864 |
| AF_1165 | V/A-type H^+^/Na^+^-transporting ATPase subunit AtpF | 487.2 | 465.2 | -0.1 | 0.777 |
| AF_1166 | V/A-type H^+^/Na^+^-transporting ATPase subunit AtpA | 694.4 | 733.4 | 0.0 | 0.993 |
| AF_1167 | V/A-type H^+^/Na^+^-transporting ATPase subunit AtpB | 517.5 | 527.9 | 0.0 | 0.907 |
| AF_1168 | V/A-type H^+^/Na^+^-transporting ATPase subunit AtpD | 403.2 | 435.1 | 0.0 | 0.968 |
|  | **Fatty acid metabolism** |  |  |  |  |
| AF_0089 | long-chain-fatty-acid--CoA ligase FadD-1 | 3.0 | 8.2 | 1.3 | 0.005 |
| AF_0200 | long-chain-fatty-acid--CoA ligase FadD-2 | 4.9 | 5.1 | -0.1 | 0.915 |
| AF_0687 | long-chain-fatty-acid--CoA ligase FadD-3 | 1.6 | 21.3 | 3.5 | 0.000 |
| AF_0840 | long-chain-fatty-acid--CoA ligase FadD-4 | 9.9 | 81.9 | 2.9 | 0.000 |
| AF_1029 | long-chain-fatty-acid--CoA ligase FadD-5 | 12.9 | 45.5 | 1.7 | 0.000 |
| AF_1510 | long-chain-fatty-acid--CoA ligase FadD-6 | 64.3 | 34.1 | -0.9 | 0.006 |
| AF_1772 | long-chain-fatty-acid--CoA ligase FadD-7 | 21.1 | 216.7 | 3.1 | 0.000 |
| AF_1932 | long-chain-fatty-acid--CoA ligase FadD-8 | 44.7 | 60.3 | 0.2 | 0.508 |
| AF_2368 | long-chain-fatty-acid--CoA ligase FadD-9 | 18.6 | 29.9 | 0.6 | 0.071 |
| AF_0199 | acyl-CoA dehydrogenase Acd-1 | 4.0 | 4.8 | 0.2 | 0.776 |
| AF_0436 | acyl-CoA dehydrogenase Acd-2 | 77.8 | 84.7 | 0.0 | 0.965 |
| AF_0498 | acyl-coA dehydrogenase Acd-3 | 668.8 | 198.4 | -1.9 | 0.000 |
| AF_0671 | acyl-CoA dehydrogenase Acd-4 | 35.4 | 67.8 | 0.8 | 0.023 |
| AF_0845 | acyl-CoA dehydrogenase Acd-5 | 6.8 | 16.2 | 1.0 | 0.010 |
| AF_0964 | acyl-CoA dehydrogenase Acd-6 | 19.3 | 184.2 | 3.2 | 0.000 |
| AF_1026 | acyl-CoA dehydrogenase Acd-7 | 21.4 | 54.8 | 1.1 | 0.002 |
| AF_1141 | acyl-CoA dehydrogenase Acd-8 | 138.4 | 80.4 | -1.0 | 0.001 |
| AF_1293 | acyl-CoA dehydrogenase Acd-9 | 89.0 | 219.8 | 1.2 | 0.000 |
| AF_2057 | acyl-CoA dehydrogenase Acd-10 | 144.9 | 180.3 | 0.1 | 0.901 |
| AF_2244 | acyl-CoA dehydrogenase Acd-11 | 30.4 | 323.5 | 3.3 | 0.000 |
| AF_2275 | acyl-CoA dehydrogenase Acd-12 | 3.9 | 5.9 | 0.5 | 0.391 |
| AF_1175 | acyl-CoA dehydrogenase, short chain-specific, AcdS | 1.8 | 13.3 | 2.8 | 0.000 |
| AF_0435 | enoyl-CoA hydratase Fad-1 | 54.9 | 67.3 | 0.2 | 0.654 |
| AF_0685 | enoyl-CoA hydratase Fad-2 | 8.9 | 17.2 | 0.7 | 0.099 |
| AF_0963 | enoyl-CoA hydratase Fad-3 | 12.2 | 148.8 | 3.5 | 0.000 |
| AF_1641 | enoyl-CoA hydratase Fad-4 | 6.1 | 8.5 | 0.3 | 0.606 |
| AF_2429 | enoyl-CoA hydratase Fad-5 | 106.5 | 114.7 | 0.0 | 0.942 |
| AF_0017 | 3-hydroxyacyl-CoA dehydrogenase Hbd-1 | 5.2 | 19.3 | 1.8 | 0.000 |
| AF_0285 | 3-hydroxyacyl-CoA dehydrogenase Hbd-2 | 493.9 | 463.4 | -0.2 | 0.405 |
| AF_0434 | 3-hydroxyacyl-CoA dehydrogenase Hbd-3 | 28.3 | 42.6 | 0.5 | 0.178 |
| AF_1025 | 3-hydroxyacyl-CoA dehydrogenase Hbd-4 | 17.6 | 38.7 | 0.9 | 0.016 |
| AF_1122 | 3-hydroxyacyl-CoA dehydrogenase Hbd-5 | 13.5 | 39.1 | 1.3 | 0.000 |
| AF_1177 | 3-hydroxyacyl-CoA dehydrogenase Hbd-6 | 3.8 | 13.3 | 1.7 | 0.000 |
| AF_1190 | 3-hydroxyacyl-CoA dehydrogenase Hbd-7 | 20.3 | 81.7 | 1.9 | 0.000 |
| AF_1206 | 3-hydroxyacyl-CoA dehydrogenase Hbd-8 | 2.6 | 6.1 | 1.1 | 0.087 |
| AF_2017 | 3-hydroxyacyl-CoA dehydrogenase Hbd-9 | 4.5 | 5.0 | 0.1 | 0.920 |
| AF_2273 | 3-hydroxyacyl-CoA dehydrogenase Hbd-10 | 75.5 | 133.5 | 0.7 | 0.016 |
| AF_0018 | 3-ketoacyl-CoA thiolase AcaB-1 | 5.9 | 16.3 | 1.4 | 0.005 |
| AF_0034 | 3-ketoacyl-CoA thiolase AcaB-2 | 4.5 | 8.0 | 0.8 | 0.147 |
| AF_0133 | 3-ketoacyl-CoA thiolase AcaB-3 | 3.9 | 5.4 | 0.4 | 0.595 |
| AF_0134 | 3-ketoacyl-CoA thiolase AcaB-4 | 3.7 | 7.5 | 0.9 | 0.086 |
| AF_0201 | 3-ketoacyl-CoA thiolase AcaB-5 | 5.3 | 6.1 | 0.1 | 0.901 |
| AF_0202 | 3-ketoacyl-CoA thiolase AcaB-6 | 4.1 | 3.5 | -0.4 | 0.622 |
| AF_0283 | 3-ketoacyl-CoA thiolase AcaB-7 | 852.7 | 600.1 | -0.7 | 0.012 |
| AF_0438 | 3-ketoacyl-CoA thiolase AcaB-8 | 51.7 | 70.1 | 0.3 | 0.391 |
| AF_0967 | 3-ketoacyl-CoA thiolase AcaB-9 | 30.3 | 272.9 | 3.1 | 0.000 |
| AF_0968 | 3-ketoacyl-CoA thiolase AcaB-10 | 19.7 | 179.6 | 3.1 | 0.000 |
| AF_1291 | 3-ketoacyl-CoA thiolase AcaB-11 | 140.7 | 317.0 | 1.0 | 0.001 |
| AF_2416 | 3-ketoacyl-CoA thiolase AcaB-12 | 62.2 | 59.1 | -0.2 | 0.526 |
| AF_1028 | 3-ketoacyl-CoA thiolase FadA-1 | 11.6 | 39.6 | 1.7 | 0.000 |
| AF_1197 | 3-ketoacyl-CoA thiolase FadA-2 | 82.4 | 88.9 | -0.1 | 0.845 |
| AF_2243 | 3-ketoacyl-CoA thiolase FadA-3 | 30.1 | 198.7 | 2.5 | 0.000 |
| AF_0033 | acyl carrier protein synthase AcaA-1 | 2.1 | 4.7 | 1.0 | 0.093 |
| AF_2415 | acyl carrier protein synthase AcaA-2 | 38.8 | 48.0 | 0.2 | 0.664 |
| AF_0991 | glutaryl-CoA dehydrogenase GcdH | 13.5 | 33.3 | 1.1 | 0.001 |
| AF_0196 | medium-chain acyl-CoA ligase AlkK-1 | 3.3 | 3.9 | 0.2 | 0.795 |
| AF_0262 | medium-chain acyl-CoA ligase AlkK-2 | 1.4 | 7.0 | 2.2 | 0.000 |
| AF_0672 | medium-chain acyl-CoA ligase AlkK-3 | 10.9 | 29.8 | 1.3 | 0.000 |
| AF_1261 | medium-chain acyl-CoA ligase AlkK-4 | 4.8 | 4.4 | -0.3 | 0.765 |
| AF_2033 | medium-chain acyl-CoA ligase AlkK-5 | 119.9 | 159.0 | 0.2 | 0.526 |
| AF_1207 | 2-deoxy-D-gluconate 3-dehydrogenase KduD | 5.3 | 12.1 | 1.1 | 0.036 |
| AF_1538 | short-chain fatty acids transporter | 2.5 | 6.9 | 1.3 | 0.012 |
| AF_1855 | 2,3-dihydrosybenzoate-AMP ligase EntE | 1.9 | 4.5 | 1.1 | 0.069 |
| AF_0287 | electron transfer flavoprotein subunit EtfA | 252.1 | 206.3 | -0.5 | 0.115 |
| AF_0286 | electron transfer flavoprotein subunit EtfB | 578.9 | 457.0 | -0.6 | 0.108 |
| AF_2215 | methylmalonyl-CoA mutase subunit McmA1 | 136.0 | 176.8 | 0.2 | 0.578 |
| AF_2219 | methylmalonyl-CoA mutase subunit McmA2 | 69.7 | 88.5 | 0.2 | 0.576 |
| AF_2217 | methylmalonyl-CoA decarboxylase subunit MmdA | 96.3 | 135.1 | 0.4 | 0.211 |
| AF_2216 | methylmalonyl-CoA decarboxylase subunit MmdC | 201.6 | 201.4 | -0.2 | 0.731 |
| AF_1288 | methylmalonyl-CoA mutase family protein | 177.1 | 437.9 | 1.1 | 0.000 |
| AF_1289 | methylmalonyl-CoA mutase-associated GTPase MeaB | 211.8 | 397.6 | 0.7 | 0.041 |
| AF_1145 | 4-hydroxybutyrate CoA transferase Cat2-1 | 18.9 | 74.7 | 1.8 | 0.000 |
| AF_0333 | 4-hydroxyphenylacetate-3-hydroxylase HpaA-1 | 5.3 | 6.6 | 0.3 | 0.771 |
| AF_0885 | 4-hydroxyphenylacetate-3-hydroxylase HpaA-2 | 42.4 | 138.2 | 1.5 | 0.000 |
| AF_1027 | 4-hydroxyphenylacetate-3-hydroxylase HpaA-3 | 6.3 | 18.8 | 1.4 | 0.000 |
| AF_1678 | putative sterol carrier protein | 21.5 | 174.0 | 2.9 | 0.000 |
| AF_1174 | putative sterol carrier protein | 2.0 | 11.9 | 2.5 | 0.000 |
| AF_0973 | putative acyl-CoA transferase/ formyl-CoA transferase | 7.5 | 94.4 | 3.5 | 0.000 |
| AF_0974 | putative acyl-CoA transferase/ formyl-CoA transferase | 7.9 | 83.6 | 3.3 | 0.000 |

**SI table 2: Genes upregulated during growth on MP versus lactate**. Values are shown for log2 fold change values above 2.5. RPKM: Reads Per Kilobase Million, *p*_adj_: adjusted p value (calculated with DESeq2 (Love *et al.*, 2014)).

| **Locus tag** | **Annotation** | **RPKM Lac** | **RPKM MP** | **log_2_ fold change** | ***p*_adj_** |
| --- | --- | --- | --- | --- | --- |
| AF_1381 | F_420_-nonreducing hydrogenase VhtG | 1.4 | 139.2 | 6.5 | 0.000 |
| AF_0007 | O-demethylase, MtoB | 68.6 | 4719.1 | 5.9 | 0.000 |
| AF_0006 | cobalamin-binding protein, MtoC | 221.7 | 13460.7 | 5.7 | 0.000 |
| AF_1380 | F_420_-nonreducing hydrogenase VhtA | 2.2 | 126.6 | 5.7 | 0.000 |
| AF_1378 | F_420_-nonreducing hydrogenase subunit VhtD-2 | 1.3 | 58.9 | 5.5 | 0.000 |
| AF_0008 | MFS transporter | 8.8 | 314.4 | 5.1 | 0.000 |
| AF_1377 | soluble heterodisulfide reductase subunit HdrA | 1.8 | 49.3 | 4.5 | 0.000 |
| AF_0009 | MtrH-like methyl transferase, MtoA | 43.6 | 1061.4 | 4.5 | 0.000 |
| AF_2435 | type I-B CRISPR-associated endonuclease Cas1 | 3.9 | 89.7 | 4.4 | 0.000 |
| AF_2183 | hypothetical protein | 6.4 | 144.3 | 4.4 | 0.000 |
| AF_1379 | quinone-reactive Ni/Fe-hydrogenase B-type cytochrome subunit HydC; Putative F420-nonreducing hydrogenase subunit VhtC | 7.0 | 170.5 | 4.4 | 0.000 |
| AF_0010 | Corrinoid activation protein, MtoD | 41.1 | 861.8 | 4.3 | 0.000 |
| AF_0468 | 2-ketoglutarate ferredoxin oxidoreductase subunit KorB | 42.2 | 835.4 | 4.2 | 0.000 |
| AF_0773 | hypothetical protein | 1.0 | 19.7 | 4.2 | 0.002 |
| AF_0011 | cobalamin-binding protein, MtoC | 17.6 | 308.8 | 4.0 | 0.000 |
| AF_0013 | MFS transporter | 1.7 | 28.5 | 4.0 | 0.000 |
| AF_2436 | CRISPR-associated protein Cas4 | 16.0 | 286.1 | 4.0 | 0.000 |
| AF_2434 | CRISPR-associated endonuclease Cas2 | 13.4 | 228.6 | 4.0 | 0.000 |
| AF_1937 | MinD/ParA family protein | 0.8 | 12.4 | 3.9 | 0.000 |
| AF_2119 | hypothetical protein | 2.7 | 41.9 | 3.9 | 0.000 |
| AF_0970 | hypothetical protein; putative transporter | 0.4 | 6.8 | 3.9 | 0.025 |
| AF_0962 | branched-chain amino acid ABC transporter BraC-3 | 5.4 | 82.2 | 3.9 | 0.000 |
| AF_1374 | F_420_-nonreducing hydrogenase subunit VhuD | 2.3 | 35.1 | 3.8 | 0.000 |
| AF_2296 | cytochrome oxidase subunit I | 12.4 | 180.7 | 3.8 | 0.000 |
| AF_0469 | 2-ketoglutarate ferredoxin oxidoreductase subunit KorA | 35.3 | 505.3 | 3.8 | 0.000 |
| AF_0824 | branched-chain amino acid ABC transporter BraE-2 | 2.5 | 33.4 | 3.7 | 0.000 |
| AF_1228 | hypothetical protein | 1.6 | 27.3 | 3.7 | 0.000 |
| AF_0052 | hypothetical protein | 2.7 | 35.1 | 3.7 | 0.000 |
| AF_2294 | MFS transporter | 1.1 | 12.8 | 3.6 | 0.000 |
| AF_0012 | O-demethylase, MtoB2 | 8.6 | 113.3 | 3.6 | 0.000 |
| AF_0368 | DUF2281 domain-containing protein | 0.0 | 3.7 | 3.6 | 0.099 |
| AF_1619 | putative sulfate exporter family transporter | 24.7 | 319.3 | 3.6 | 0.000 |
| AF_0825 | branched-chain amino acid ABC transporter BraD-2 | 2.9 | 35.2 | 3.6 | 0.000 |
| AF_0973 | putative acyl-CoA transferase/formyl-CoA transferase | 7.5 | 94.4 | 3.5 | 0.000 |
| AF_1118 | hypothetical protein | 1.8 | 21.4 | 3.5 | 0.000 |
| AF_0963 | enoyl-CoA hydratase Fad-3 | 12.2 | 148.8 | 3.5 | 0.000 |
| AF_2295 | aspartate aminotransferase family protein | 4.5 | 54.3 | 3.5 | 0.000 |
| AF_0687 | long-chain-fatty-acid--CoA ligase FadD-3 | 1.6 | 21.3 | 3.5 | 0.000 |
| AF_0762 | hypothetical protein | 1.1 | 12.1 | 3.4 | 0.000 |
| AF_0467 | TAXI family TRAP transporter solute-binding subunit | 23.6 | 259.2 | 3.4 | 0.000 |
| AF_0763 | hypothetical protein | 7.1 | 86.5 | 3.4 | 0.000 |
| AF_0816 | putative V-type ATP synthase subunit J | 2.8 | 31.3 | 3.4 | 0.000 |
| AF_0806 | L-lactate permease LctP | 34.4 | 331.6 | 3.4 | NA |
| AF_0823 | branched-chain amino acid ABC transporter BraG-2 | 6.1 | 65.3 | 3.4 | 0.000 |
| AF_0470 | 2-ketoglutarate ferredoxin oxidoreductase subunit KorD | 53.5 | 552.8 | 3.3 | 0.000 |
| AF_0761 | hypothetical protein | 8.6 | 98.0 | 3.3 | 0.000 |
| AF_0312 | class I SAM-dependent methyltransferase | 0.2 | 2.6 | 3.3 | 0.038 |
| AF_0015 | proline permease | 0.5 | 5.3 | 3.3 | 0.000 |
| AF_0974 | putative acyl-CoA transferase/formyl-CoA transferase | 7.9 | 83.6 | 3.3 | 0.000 |
| AF_2244 | acyl-CoA dehydrogenase Acd-11 | 30.4 | 323.5 | 3.3 | 0.000 |
| AF_1005 | ABC transporter permease | 0.6 | 6.1 | 3.2 | 0.000 |
| AF_2297 | cytochrome ubiquinol oxidase subunit I | 19.9 | 199.8 | 3.2 | 0.000 |
| AF_2121 | hypothetical protein | 6.8 | 65.8 | 3.2 | 0.000 |
| AF_1373 | F_420_-nonreducing hydrogenase subunit VhuG | 3.8 | 36.8 | 3.2 | 0.000 |
| AF_0124 | sulfite exporter TauE/SafE family protein | 2.0 | 20.0 | 3.2 | 0.000 |
| AF_2120 | hypothetical protein | 4.8 | 46.4 | 3.2 | 0.000 |
| AF_1618 | putative sulfate exporter family transporter | 72.1 | 751.9 | 3.2 | 0.000 |
| AF_1617 | E3 ubiquitin-protein ligase | 58.1 | 583.6 | 3.2 | 0.000 |
| AF_0964 | acyl-CoA dehydrogenase Acd-6 | 19.3 | 184.2 | 3.2 | 0.000 |
| AF_0471 | 2-ketoglutarate ferredoxin oxidoreductase subunit KorG | 17.0 | 168.0 | 3.2 | 0.000 |
| AF_1772 | long-chain-fatty-acid--CoA ligase FadD-7 | 21.1 | 216.7 | 3.1 | 0.000 |
| AF_2409 | 4Fe-4S dicluster domain-containing protein | 2.0 | 18.3 | 3.1 | 0.000 |
| AF_0822 | branched-chain amino acid ABC transporter BraF-2 | 6.5 | 61.1 | 3.1 | 0.000 |
| AF_1301 | ABC transporter permease | 1.0 | 8.5 | 3.1 | 0.052 |
| AF_0664 | DUF22 domain-containing protein | 19.9 | 185.7 | 3.1 | 0.000 |
| AF_1679 | putative V-type ATP synthase subunit J | 4.1 | 37.1 | 3.1 | 0.000 |
| AF_0968 | 3-ketoacyl-CoA thiolase AcaB-10 | 19.7 | 179.6 | 3.1 | 0.000 |
| AF_0967 | 3-ketoacyl-CoA thiolase AcaB-9 | 30.3 | 272.9 | 3.1 | 0.000 |
| AF_0615 | GtrA family protein | 0.0 | 1.1 | 3.0 | 0.231 |
| AF_0959 | branched-chain amino acid ABC transporter BraF-3 | 5.5 | 46.8 | 3.0 | 0.000 |
| AF_1376 | soluble heterodisulfide reductase subunit HdrC | 1.4 | 12.6 | 3.0 | 0.000 |
| AF_0976 | acetyl-CoA synthetase Acs-5 | 15.1 | 130.8 | 3.0 | 0.000 |
| AF_2122 | hypothetical protein | 10.0 | 80.1 | 3.0 | 0.000 |
| AF_1226 | hypothetical protein | 11.9 | 105.2 | 2.9 | 0.000 |
| AF_0906 | hydantoinase/oxoprolinase family protein | 10.5 | 86.9 | 2.9 | 0.000 |
| AF_0975 | acetyl-CoA synthetase Acs-4 | 18.3 | 147.6 | 2.9 | 0.000 |
| AF_1678 | putative sterol carrier protein | 21.5 | 174.0 | 2.9 | 0.000 |
| AF_0840 | long-chain-fatty-acid--CoA ligase FadD-4 | 9.9 | 81.9 | 2.9 | 0.000 |
| AF_0131 | nitroreductase family protein | 3.5 | 28.4 | 2.8 | 0.000 |
| AF_0026 | TrmB family transcriptional regulator | 5.0 | 38.5 | 2.8 | 0.000 |
| AF_1375 | soluble heterodisulfide reductase subunit HdrB | 5.9 | 47.1 | 2.8 | 0.000 |
| AF_0965 | Zn-ribbon domain-containing OB-fold protein | 25.5 | 194.2 | 2.8 | 0.000 |
| AF_2374 | TolB family protein | 0.6 | 4.6 | 2.8 | 0.030 |
| AF_1175 | acyl-CoA dehydrogenase, short chain-specific, AcdS | 1.8 | 13.3 | 2.8 | 0.000 |
| AF_1227 | hypothetical protein; putative methionine synthase | 10.8 | 89.5 | 2.8 | 0.000 |
| AF_0472 | phosphate uptake regulator PhoU | 2.3 | 16.7 | 2.8 | 0.000 |
| AF_0123 | hypothetical protein | 1.8 | 13.4 | 2.8 | 0.001 |
| AF_0121 | DUF22 domain-containing protein | 6.0 | 48.7 | 2.8 | 0.000 |
| AF_1173 | hypothetical protein | 2.7 | 20.6 | 2.8 | 0.000 |
| AF_0966 | Zn-ribbon domain-containing OB-fold protein | 47.3 | 350.0 | 2.8 | 0.000 |
| AF_1949 | hypothetical protein | 2.9 | 20.2 | 2.7 | 0.000 |
| AF_0337 | histone family protein | 294.7 | 2496.3 | 2.7 | 0.000 |
| AF_0969 | cation acetate symporter (acyP) | 1.9 | 13.5 | 2.7 | 0.000 |
| AF_1372 | F_420_-nonreducing hydrogenase subunit VhuA | 4.6 | 30.9 | 2.7 | 0.000 |
| AF_1225 | hypothetical protein | 7.9 | 56.2 | 2.7 | 0.000 |
| AF_0366 | acetyl-CoA synthetase Acs-2 | 4.1 | 28.0 | 2.6 | 0.000 |
| AF_0772 | hypothetical protein | 7.1 | 45.1 | 2.6 | 0.000 |
| AF_0016 | hypothetical protein; cytochrome c | 1.2 | 7.4 | 2.6 | 0.003 |
| AF_1428 | DUF2193 domain-containing protein | 14.7 | 97.5 | 2.6 | 0.000 |
| AF_2281 | aldehyde ferredoxin oxidoreductase Aor-4 | 32.2 | 208.0 | 2.6 | 0.000 |
| AF_1208 | cupin domain-containing protein | 1.4 | 8.2 | 2.6 | 0.017 |
| AF_2243 | 3-ketoacyl-CoA thiolase FadA-3 | 30.1 | 198.7 | 2.5 | 0.000 |
| AF_2357 | transglutaminase domain-containing protein | 1.1 | 7.4 | 2.5 | 0.000 |
| AF_0220 | pyruvate carboxylase PycA | 31.2 | 169.6 | 2.5 | 0.000 |
| AF_0140 | ubiquinone/menaquinone biosynthesis methyltransferase UbiE | 0.0 | 0.5 | 2.5 | 0.459 |
| AF_1174 | putative sterol carrier protein | 2.0 | 11.9 | 2.5 | 0.000 |

**SI table 3: Genes downregulated during growth on MP versus lactate**. Values are shown for log2 fold change values below -1.5. RPKM: Reads Per Kilobase Million, *p*_adj_: adjusted *p* value (calculated with DESeq2 (Love *et al.*, 2014)).

| **Locus tag** | **Annotation** | **RPKM Lac** | **RPKM MP** | **log_2_ fold change** | ***p*_adj_** |
| --- | --- | --- | --- | --- | --- |
| AF_0141 | hypothetical protein | 168.2 | 5.9 | -5.2 | 0.000 |
| AF_0977 | ammonium transporter Amt-1 | 178.3 | 7.9 | -4.3 | 0.000 |
| AF_0949 | type I glutamate--ammonia ligase GlnA | 420.1 | 26.4 | -4.0 | 0.000 |
| AF_0978 | P-II family nitrogen regulator GlnB-1 | 360.3 | 19.8 | -4.0 | 0.000 |
| AF_1745 | CopG family transcriptional regulator | 31.3 | 1.9 | -3.9 | 0.000 |
| AF_0192 | heavy metal translocating P-type ATPase | 22.3 | 1.9 | -3.8 | 0.000 |
| AF_1204 | hypothetical protein | 3.2 | 0.0 | -3.5 | 0.200 |
| AF_1148 | Lrp/AsnC family transcriptional regulator | 149.9 | 20.0 | -3.0 | 0.000 |
| AF_0245 | iron (metal) dependent repressor DtxR | 154.7 | 22.5 | -3.0 | 0.000 |
| AF_1356 | PstS family phosphate ABC transporter | 100.8 | 14.9 | -3.0 | 0.000 |
| AF_0092 | ATP-binding cassette domain-containing protein | 74.1 | 11.7 | -2.9 | 0.000 |
| AF_1749 | ammonium transporter | 11.5 | 1.5 | -2.9 | 0.000 |
| AF_1010 | 4Fe-4S dicluster domain-containing protein | 1.6 | 0.0 | -2.9 | 0.344 |
| AF_0246 | ferrous iron transport protein FeoB | 192.0 | 34.3 | -2.8 | 0.000 |
| AF_1848 | HTH DNA binding domain protein | 11.5 | 2.3 | -2.7 | 0.032 |
| AF_0614 | nucleotidyltransferase domain-containing domain | 2.7 | 0.5 | -2.6 | 0.170 |
| AF_0191 | heavy metal translocating P-type ATPase | 9.1 | 1.7 | -2.6 | 0.039 |
| AF_1359 | phosphate ABC transporter ATP-binding protein PstB | 69.2 | 13.0 | -2.5 | 0.000 |
| AF_2212 | antitoxin family protein | 1.1 | 0.0 | -2.5 | 0.513 |
| AF_0718 | metallo-beta-lactamase family protein | 5.4 | 0.9 | -2.5 | 0.206 |
| AF_0094 | tungstate ABC transporter substrate-binding protein WtpA | 83.9 | 19.6 | -2.4 | 0.000 |
| AF_0142 | cytochrome C oxidase subunit II | 3.2 | 0.8 | -2.4 | 0.099 |
| AF_0189 | hypothetical protein | 812.4 | 224.7 | -2.3 | 0.024 |
| AF_0931 | molybdenum cofactor biosynthesis protein MoeA-1 | 244.7 | 61.2 | -2.3 | 0.000 |
| AF_1565 | peptidase domain-containing protein | 75.9 | 16.6 | -2.3 | 0.000 |
| AF_0056 | DNA-directed RNA polymerase subunit P | 21.4 | 5.2 | -2.2 | 0.023 |
| AF_0904 | M20 family metallo-hydrolase | 329.0 | 85.4 | -2.1 | 0.000 |
| AF_0093 | ABC transporter permease | 27.4 | 7.1 | -2.1 | 0.000 |
| AF_1312 | outer membrane lipoprotein-sorting protein | 152.6 | 43.7 | -2.1 | 0.000 |
| AF_0930 | molybdopterin molybdotransferase MoeA | 86.2 | 25.2 | -2.1 | 0.000 |
| AF_1853 | winged helix-turn-helix transcriptional regulator | 26.1 | 6.4 | -2.1 | 0.000 |
| AF_0399 | hypothetical protein | 6.5 | 1.7 | -2.1 | 0.004 |
| AF_1480 | PilT protein domain protein | 1.1 | 0.0 | -2.0 | 0.679 |
| AF_2172 | glycine betaine transporter periplasmic subunit | 110.9 | 30.2 | -2.0 | 0.000 |
| AF_1564 | ArsR family transcriptional regulator | 54.4 | 13.5 | -2.0 | 0.000 |
| AF_2175 | hypothetical protein; putative exonuclease V | 30.8 | 9.2 | -2.0 | 0.000 |
| AF_0903 | winged helix-turn-helix transcriptional regulator | 313.4 | 91.4 | -2.0 | 0.000 |
| AF_1393 | branched-chain amino acid ABC transporter permease | 18.4 | 4.9 | -2.0 | 0.000 |
| AF_1358 | phosphate ABC transporter permease PstA | 20.2 | 5.6 | -2.0 | 0.001 |
| AF_1357 | phosphate ABC transporter permease subunit PstC | 17.9 | 5.0 | -1.9 | 0.000 |
| AF_1762 | enoyl-CoA hydratase | 1.5 | 0.3 | -1.9 | 0.515 |
| AF_0498 | acyl-coA dehydrogenase Acd-3 | 668.8 | 198.4 | -1.9 | 0.000 |
| AF_0148 | hypothetical protein | 7.1 | 2.2 | -1.9 | 0.094 |
| AF_1254 | GNAT family N-acetyltransferase | 1022.0 | 329.5 | -1.9 | 0.000 |
| AF_0396 | ArsR family transcriptional regulator | 9.0 | 2.6 | -1.8 | 0.001 |
| AF_1699 | pyruvate ferredoxin oxidoreductase subunit PorG | 1097.3 | 364.8 | -1.8 | 0.000 |
| AF_0561 | ferrous iron transport protein B | 31.4 | 10.0 | -1.8 | 0.000 |
| AF_0145 | hypothetical protein | 0.8 | 0.2 | -1.8 | 0.576 |
| AF_1186 | L-aspartate semialdehyde sulfurtransferase | 62.4 | 18.6 | -1.8 | 0.000 |
| AF_1597 | hypothetical protein | 19.4 | 6.4 | -1.8 | 0.024 |
| AF_1821 | hypothetical protein | 21.6 | 6.7 | -1.8 | 0.007 |
| AF_1390 | ABC transporter ATP-binding protein | 180.5 | 62.6 | -1.7 | 0.000 |
| AF_1700 | pyruvate ferredoxin oxidoreductase subunit PorD | 178.7 | 61.0 | -1.7 | 0.000 |
| AF_1394 | hypothetical protein | 40.1 | 13.7 | -1.7 | 0.000 |
| AF_1722 | DNA polymerase II large subunit | 343.8 | 123.6 | -1.6 | 0.000 |
| AF_2182 | transcriptional regulator | 7.9 | 2.9 | -1.6 | 0.091 |
| AF_2003 | molybdopterin-guanine dinucleotide biosynthesis protein B | 71.9 | 26.7 | -1.6 | 0.001 |
| AF_2117 | DNA-3-methyladenine glycosylase 2 family protein | 53.8 | 19.2 | -1.6 | 0.000 |
| AF_1511 | GNAT family N-acetyltransferase | 151.3 | 50.9 | -1.6 | 0.000 |
| AF_1360 | phosphate signaling complex protein PhoU | 98.2 | 35.2 | -1.6 | 0.000 |
| AF_0729 | energy-coupling factor ABC transporter substrate-binding protein | 1.0 | 0.2 | -1.6 | 0.660 |
| AF_1425 | cofactor-independent phosphoglycerate mutase | 228.4 | 84.0 | -1.6 | 0.000 |
| AF_0143 | cytochrome c oxidase subunit II | 2.6 | 1.0 | -1.6 | 0.043 |
| AF_1333 | 50S ribosomal protein L44e | 1025.1 | 410.1 | -1.6 | 0.000 |
| AF_1514 | AF1514 family protein | 22.1 | 8.3 | -1.6 | 0.013 |
| AF_1342 | AMP phosphorylase | 397.0 | 143.9 | -1.6 | 0.000 |
| AF_1610 | S-adenosylmethionine decarboxylase | 179.0 | 72.3 | -1.6 | 0.000 |
| AF_1750 | P-II family nitrogen regulator | 50.5 | 17.7 | -1.5 | 0.000 |
| AF_0155 | 4Fe-4S ferredoxin | 307.0 | 124.8 | -1.5 | 0.203 |
| AF_1341 | AMP phosphorylase | 311.1 | 114.6 | -1.5 | 0.000 |
| AF_1391 | ABC transporter substrate-binding protein | 358.7 | 146.2 | -1.5 | 0.000 |
| AF_1242 | hydroxymethylbilane synthase HemC | 183.6 | 73.8 | -1.5 | 0.000 |
| AF_1102 | hypothetical protein | 124.1 | 49.2 | -1.5 | 0.000 |
| AF_1462 | FAD/NAD(P)-binding:oxidoreductase | 2.8 | 1.1 | -1.5 | 0.209 |
| AF_0301 | DUF2281 domain-containing protein | 6.0 | 2.4 | -1.5 | 0.289 |
| AF_1388 | IGHMBP2 family helicase | 39.6 | 15.9 | -1.5 | 0.000 |
| AF_1854 | acetyl-CoA hydrolase/transferase family protein | 479.9 | 201.0 | -1.5 | 0.000 |
| AF_1361 | low molecular weight phosphatase family protein | 139.0 | 58.4 | -1.5 | 0.000 |
| AF_0165 | sulfurtransferase TusA family protein | 6.8 | 2.8 | -1.5 | 0.224 |
| AF_0954 | Glutamate synthase, alpha subunit domain | 356.0 | 145.2 | -1.5 | 0.000 |
| AF_2240 | Rubrerythrin | 9.4 | 3.8 | -1.5 | 0.200 |
